# Identification and semisynthesis of (−)-anisomelic acid as oral agent against SARS-CoV-2 in mice

**DOI:** 10.1093/nsr/nwac176

**Published:** 2022-08-26

**Authors:** Hai-Xin Yu, Nan Zheng, Chi-Tai Yeh, Chien-Ming Lee, Qi Zhang, Wen-Lv Zheng, Qing Chang, Yuan-He Li, Yu-Jun Li, Gui-Zhen Wu, Jun-Min Quan, Lin-Qi Zhang, Yew-Min Tzeng, Zhen Yang

**Affiliations:** Laboratory of Chemical Genomics, School of Chemical Biology and Biotechnology, Peking University Shenzhen Graduate School, Shenzhen 518055; Laboratory of Chemical Genomics, School of Chemical Biology and Biotechnology, Peking University Shenzhen Graduate School, Shenzhen 518055; Department of Medicinal Research and Education, Taipei Medical University-Shuang Ho Hospital, New Taipei City 23561; Department of Applied Science, Taitung University, Taitung 95092; Center for Global Health and Infectious Diseases, Comprehensive AIDS Research Center, and Beijing Advanced Innovation Center for Structural Biology, School of Medicine, Tsinghua University, Beijing 100084; Laboratory of Chemical Genomics, School of Chemical Biology and Biotechnology, Peking University Shenzhen Graduate School, Shenzhen 518055; Lanzhou Institute of Separation Science, Lanzhou 730013; State Key Laboratory of Bioorganic Chemistry and Molecular Engineering of Ministry of Education and Beijing National Laboratory for Molecular Science (BNLMS), College of Chemistry and Molecular Engineering, Peking University, Beijing 100871; Shenzhen Bay Laboratory, Shenzhen 518055; NHC Key Laboratory of Biosafety, National Institute for Viral Disease Control and Prevention, Chinese Center for Disease Control and Prevention, Beijing 102206; Laboratory of Chemical Genomics, School of Chemical Biology and Biotechnology, Peking University Shenzhen Graduate School, Shenzhen 518055; Center for Global Health and Infectious Diseases, Comprehensive AIDS Research Center, and Beijing Advanced Innovation Center for Structural Biology, School of Medicine, Tsinghua University, Beijing 100084; Department of Applied Science, Taitung University, Taitung 95092; Department of Applied Chemistry, Chaoyang University of Technology, Taichung 41349; Laboratory of Chemical Genomics, School of Chemical Biology and Biotechnology, Peking University Shenzhen Graduate School, Shenzhen 518055; State Key Laboratory of Bioorganic Chemistry and Molecular Engineering of Ministry of Education and Beijing National Laboratory for Molecular Science (BNLMS), College of Chemistry and Molecular Engineering, Peking University, Beijing 100871; Shenzhen Bay Laboratory, Shenzhen 518055

**Keywords:** identification, enantioselective semisynthesis, (−)-anisomelic acid, oral agent, anti-SARS-CoV-2

## Abstract

(−)-Anisomelic acid, isolated from *Anisomeles indica* (L.) Kuntze (Labiatae) leaves, is a macrocyclic cembranolide with a *trans*-fused α-methylene-γ-lactone motif. Anisomelic acid effectively inhibits SARS-CoV-2 replication and viral-induced cytopathic effects with an EC_50_ of 1.1 and 4.3 μM, respectively. Challenge studies of SARS-CoV-2-infected K18-hACE2 mice showed that oral administration of anisomelic acid and subcutaneous dosing of remdesivir can both reduce the viral titers in the lung tissue at the same level. To facilitate drug discovery, we used a semisynthetic approach to shorten the project timelines. The enantioselective semisynthesis of anisomelic acid from the naturally enriched and commercially available starting material (+)-costunolide was achieved in five steps with a 27% overall yield. The developed chemistry provides opportunities for developing anisomelic-acid-based novel ligands for selectively targeting proteins involved in viral infections.

## Teaser

The natural product (−)-anisomelic acid has been identified as an orally available antiviral agent against SARS-CoV-2.

## INTRODUCTION

The coronavirus disease 2019 (COVID-19) pandemic poses serious threats to global public health and places severe economic strains on the world's economy [1]. Effective vaccines against COVID-19 can offer a long-term control strategy, but the ability of SARS-CoV-2 mutations to escape acquired immunity is a growing cause for concern [2]. Small-molecule-based antiviral agents [3] can help to address the global threat of COVID-19. These agents, which can be easily distributed to patients and rapidly manufactured from simple and readily available commodity reagents, have a low tendency to induce drug-resistant mutations. Various drug candidates have been evaluated as treatment options [4] and remdesivir [5,6], molnupiravir [7] and paxlovid [8] have been approved under an emergency-use authorization. The identification of broadly effective antivirals with favorable benefit/risk profiles could address the serious need for the treatment of diseases caused by pathogenic coronaviruses.

Natural products have been spotlighted as potential sources of new leads for the development of therapeutic agents [9] because of their unparalleled structural diversity and complexity. *Anisomeles indica* (L.) Kuntze (Labiatae) is found throughout the southern and tropical regions of Asia. In traditional Chinese medicine, *A. indica* is commonly known as ‘yu-chen-tsao’ or ‘fang-feng-cao’. The dried whole *A. indica* plant has traditionally been used for the treatment of inflammation [10], pain [11] and influenza [12].

## RESULTS AND DISCUSSION

**Figure 1.**
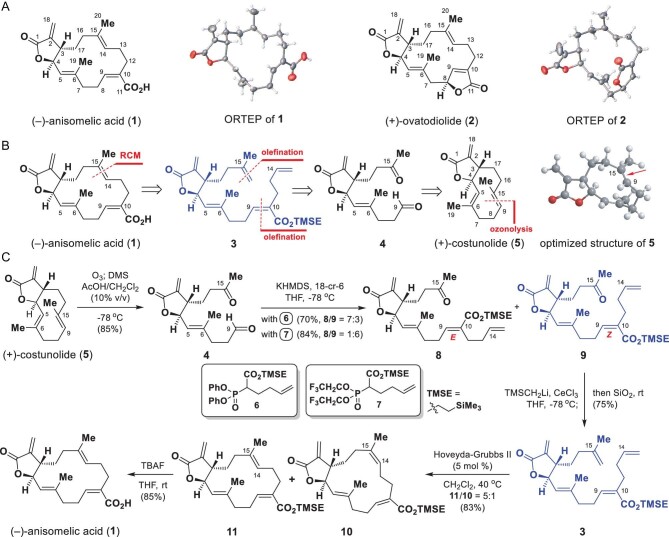
Semisynthesis of (−)-anisomelic acid (**1**). (A) Structures and ORTEP (Oak Ridge thermal-ellipsoid plot) diagrams of (−)-anisomelic acid (**1**) and (+)-ovatodiolide (**2**). (B) Retrosynthetic analysis of (−)-anisomelic acid (**1**). (C) Semisynthesis of (−)-anisomelic acid (**1**). DMS, dimethyl sulfide; TBAF, tetrabutylammonium fluoride; THF, tetrahydrofuran; TMSE, trimethylsilylethyl.

In the early stages of the virus outbreak in 2020, we were inspired by the traditional use of *A. indica* in the treatment of infectious diseases to identify the active ingredients in this herbal medicine and investigated their use in the treatment of COVID-19. Cytopathic effect (CPE) assays showed that (−)-anisomelic acid and (+)-ovatodiolide (**1** and **2**, Fig. 1A) have anti-SARS-CoV-2 activities, with potencies of IC_50_ = 4.3 and 3.0 μM, respectively. In addition, challenge studies using a K18-hACE2 mouse model of SARS-CoV-2 showed that the reduction in the viral titers achieved with **1** and **2** were comparable to that obtained with remdesivir in oral administration experiments. This indicates that **1** and **2** are potential lead compounds for the development of antiviral agents.

Structurally, **1** and **2** belong to a class of naturally occurring macrocyclic cembranolides [13] and share a basic structure that consists of a 14-membered macrocyclic core with three trisubstituted olefins and a *trans*-fused α-methylene-γ-lactone. The only difference between these two compounds is that the carboxyl motif in **1** corresponds to a lactone in **2**. In 1975, **1** and **2** were isolated from a different plant, i.e. *Anisomeles malabarica*, and identified [14], but their biological activities were not evaluated at that time.

The majority of currently reported antiviral agents are derivatives of nucleic acids and amino acids, derived from the substrates of viral enzymes [4], and therefore the development of antiviral agents based on the core structures of **1** and **2** could afford an alternative scaffold for antiviral drug discovery. Although compound **1** can be extracted from the plant *Artemisia annua*, it only constitutes <0.02% m/m of the plant biomass. The supplies of plant-derived **1** are unstable and inadequate for future drug development. Given the potential demand for **1**, the development of a short and sustainable synthesis from simple raw materials, which would minimize the time needed for its manufacture and supply, is important.

The total syntheses of germacranolides have been the focus of intense research for decades, but only a few approaches to the syntheses of racemic germacranolides have been published [15]. In 1987, Marshall's group reported the total synthesis of (−)-anisomelic acid (**1**) in racemic form. The synthesis requires 21 steps and the overall yield is ∼1% [16]. Structurally, **1** has an unusual 5/14 bicyclic ring system featuring a *trans*-fused α-methylene-γ-lactone moiety. This type of lactone motif is present in naturally enriched and renewable germacranolides [17] and therefore we decided to develop a semisynthetic approach to preparing optically pure **1** from naturally occurring germacranolides.

In this context, (+)-costunolide (**5**, Fig. 1B) attracted our attention because it is naturally abundant and has a *trans*-fused α-methylene-γ-lactone motif and has frequently been used as a starting material for the semisynthesis of biologically important molecules [18,19]. Figure 1B shows our retrosynthetic analysis. The macrocyclic core in the target molecule **1** can be formed regioselectively from diene **3** via a Grubbs’s [20] ring-closing metathesis (RCM) reaction as the key step. The diene **3** can be generated via consecutive olefinations from ketoaldehyde **4**, which can be synthesized from (+)-costunolide (**5**) via a regioselective ozonolysis reaction. Although the proposed regioselective ozonolysis is challenging, we envisaged that the electron-rich nature [21] of the C9=C15 double bond in **5** would improve its accessibility to ozone (see the 3D structure of **5** in Fig. 1B), which would make this reaction feasible. The built-in chirality in **5** could be transferred to **1** to achieve a chiral pool strategy [22], which would enable the construction of (+)-ovatodiolide (**2**) from the same chiral compound.

Our synthesis began with the preparation of ketoaldehyde **4** from (+)-costunolide (**5**) via ozonolysis (Fig. 1C). Initial attempts to achieve ozonolysis of **5** failed to afford **4** and serious decomposition was observed. However, when the reaction was performed at −78°C in CH_2_Cl_2_ with acetic acid (10%) as a co-solvent, **4** was obtained in 85% yield. We then moved on to the synthesis of ketone **9**. Initially, a Honer–Wadsworth–Emmons reaction of **4** with **6** in the presence of potassium hexamethyldisilazide (KHMDS) and 18-crown-6 ether (18-C-6) gave the more stable [23] (*E*)-olefin **8** as the major isomer in 49% yield, together with **9** as a minor isomer (21%). To improve the selectivity profile, we prepared the Still–Gennari electrophilic reagent **7** [24] and reacted it with **4** in the presence of  KHMDS and 18-crown-6 ether; (*Z*)-olefin **9** was the predominant product (72% yield) and **8** was formed in 12% yield.

The syntheses of diene **3** using various standard methods, such as the Wittig reaction and Tebbe reaction, were investigated, but synthetically useful yields of **3** were not obtained. Use of a modified Peterson olefination reaction [25] solved this problem and diene **3** was obtained in 75% yield when ketone **9** was reacted with excess LiCH_2_Si(CH_3_)_3_/CeCl_3_ with subsequent silica-gel-mediated elimination. We then evaluated the feasibility of macrocyclic ring formation via the proposed RCM reaction (Fig. 1C). Several RCM reagents were profiled. In the presence of the Hoveyda–Grubbs II catalyst, the desired annulation proceeded smoothly under the optimal conditions, i.e. 0.0002 M diene **3** in CH_2_Cl_2_ with 0.05 equiv. of the catalyst. This method gave compounds **10** and **11** in a combined yield of 83% at a **10** : **11** ratio of 1 : 5. Conventional treatment of **11** with TBAF in THF at 25°C gave **1** in 85% yield. The NMR spectroscopic data and optical rotation of **1** were consistent with the data reported for natural (−)-anisomelic acid; the identity of **1** was unambiguously confirmed using single-crystal X-ray analysis. We therefore achieved, for the first time, an enantioselective semisynthesis of (−)-anisomelic acid (**1**) from (+)-costunolide (**5**) in five steps with a 27% overall yield.

Our successful synthesis of (−)-anisomelic acid (**1**) encouraged us to explore the therapeutic potential of **1** and (+)-ovatodiolide (**2**) in the treatment of COVID-19.

**Figure 2.**
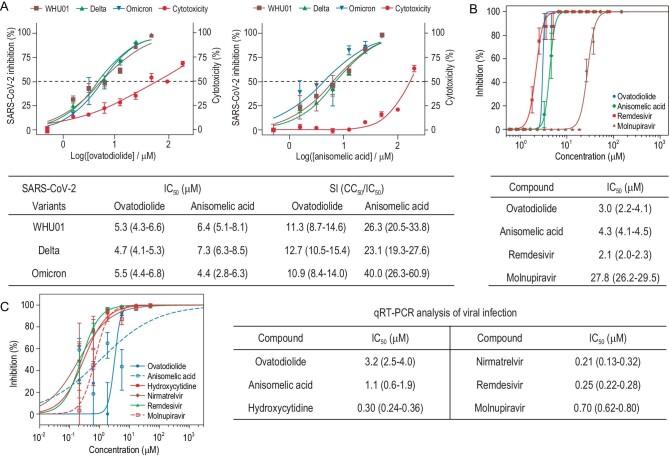
Antiviral activities of (+)-ovatodiolide and (−)-anisomelic acid against SARS-CoV-2 *in vitro*. (A) Antiviral activities of (−)-anisomelic acid and (+)-ovatodiolide against SARS-CoV-2 spike pseudotyped viruses. HeLa-hACE2 cells were treated with these two compounds at the indicated doses and infected with the reporter SARS-CoV-2 pseudotyped virus for 48 h. The titer percentage was determined using *Gaussia* luciferase luminescence flash assay. Cytotoxicity (red) was determined using the CCK-8 test. Data are expressed as the mean ± SD of three independent experiments. Numbers in parentheses are 95% confidence intervals. (B) Antivirus activities of compounds against live SARS-CoV-2 virus by cytopathic effect (CPE) assay. Vero E6 cells were treated with compounds at indicated doses and infected with 100 TCID50 live SARS-CoV-2 viruses (F13 strain) for 4 days. Inhibition was measured as the reciprocal of the serum dilution for 50% neutralization of viral infection. Numbers in parentheses are 95% confidence intervals. (C) Antivirus activities of compounds against live SARS-CoV-2 virus by qRT-PCR. Calu-3 cells treated with compounds at indicated doses and infected with live SARS-CoV-2 viruses (Delta) at a MOI = 0.05 for 48 h. Viral yield in the cell supernatant was then quantified using qRT-PCR. Data are expressed as mean ± SD of three independent experiments. Numbers in parentheses are 95% confidence intervals.

We first tested their abilities to inhibit SARS-CoV-2 infection by using a previously established SARS-CoV-2 pseudovirus neutralization assay [26]. Both **1** and **2** showed moderate anti-SARS-CoV-2 activities, with IC_50_ values of 6.4 and 5.3 μM, respectively. Their cytotoxicities in HeLa-hACE2 cells were examined in parallel with their antiviral activities. The CC_50_ of **1** is 167.2 μM; **2** had a higher cytotoxicity, with a CC_50_ value of 59.3 μM (Fig. 2A). Because of their increased transmissibilities and virulence, and the possibility of decreased effectiveness toward diagnostics, vaccines and therapeutics, newly emerged SARS-CoV-2 mutant variants pose a potential threat to public health. We therefore used pseudovirus neutralization assays [27] to assess the antiviral activities of **1** and **2** against recently prevailing SARS-CoV-2 variants such as Delta and Omicron. Both compounds showed similar antiviral potencies against SARS-CoV-2 variants compared with the wild-type strain WHU01 (Fig. 2A).

We next assessed the inhibitory activities of both natural products against the CPE of the SARS-CoV-2 virus F13 strain on Vero E6 cells [28]; remdesivir and molnupiravir were used as positive controls. Live-virus assays were performed in a BSL-3 laboratory at the National Institute for Viral Disease Control and Prevention, China CDC. The results indicated that (−)-anisomelic acid and (+)-ovatodiolide both show low micromolar activities, with IC_50_ values of 4.3 and 3.0 μM, respectively. Remdesivir showed a comparable potency, with an IC_50_ of 2.1 μM, which agrees with the previously reported value [29], whereas molnupiravir exhibited much lower potency, with an IC_50_ of 27.8 μM (Fig. 2B). We further evaluate the antivirus activities of (−)-anisomelic acid and (+)-ovatodiolide against the infection of the SARS-CoV-2 Delta variant on the human airway cell line Calu-3 (Fig. 2C). The results showed that (−)-anisomelic acid and (+)-ovatodiolide had similar activities to those in the CPE reduction assay, with IC_50_ values of 3.2 and 1.1 μM, respectively, whereas remdesivir, molnupiravir, β-D-*N*^4^-hydroxycytidine (NHC) and nirmatrelvir (PF-07321332) showed relatively higher antivirus activities compared to (−)-anisomelic acid and (+)-ovatodiolide. Notably, molupiravir (a prodrug of NHC) showed much higher antiviral activity in human Calu-3 cells compared to its anti-CPE activity in monkey Vero E6 cells, which was consistent with the previous observation that the potencies of antiviral drugs were improved in human cells, such as Calu-3 and Caco-2 cells, compared to Vero E6 cells [30].

We next examined the ability of (−)-anisomelic acid to inhibit potential targets in the viral infection cycle. The pseudovirus assay separates the viral entry from other steps of the viral infection cycle [31]. The data presented above indicate that (−)-anisomelic acid may interfere with the entry process (Fig. 2A)and B). The SARS-CoV-2 entry process involves several steps, namely receptor binding, proteolytic processing of the spike protein and membrane fusion [32]. We established *in vitro* biochemical assays to evaluate the potential targets of (−)-anisomelic acid and the reliability of the assays was confirmed by the reported positive control (Supplementary Fig. S4).

**Figure 3.**
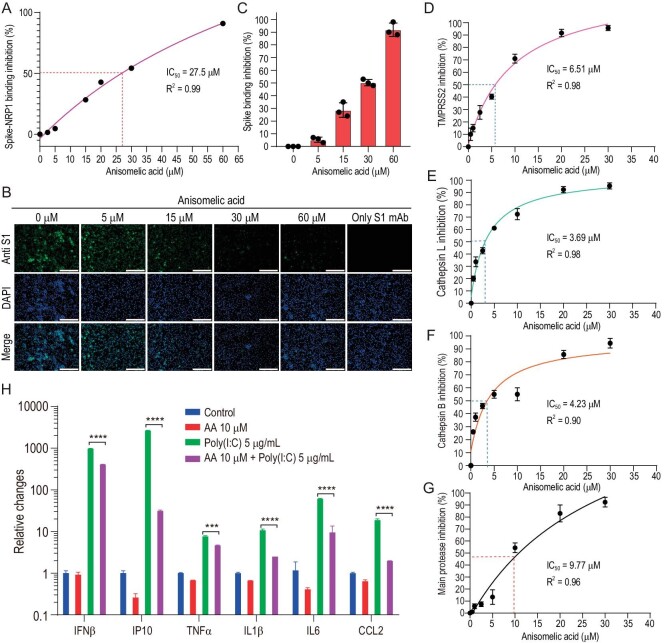
(−)-Anisomelic acid inhibition of multiple targets in viral infection cycle. (A)–(C) (−)-Anisomelic acid gave dose-dependent inhibition of binding of the viral spike protein with neuropilin-1 and NRP1-positive Huh7 cell line. Scale bar, 200 μM. (D)–(F) (−)-Anisomelic acid inhibited the activity of TMPRSS2 (D), cathepsin L (E) and cathepsin B (F) in a dose-dependent manner. (G) Dose–response curve for main protease inhibition by (−)-anisomelic acid. (H) Inhibition of mRNA expression of inflammatory cytokine of THP-1 cell line with poly(I : C) transfection by (−)-anisomelic acid.

We first profiled the interaction between (−)-anisomelic acid and ACE2 (the primary receptor of SARS-CoV-2 virus) but no binding was observed. We then tested the inhibitory effect of (−)-anisomelic acid against binding of the viral spike protein with neuropilin-1 (NRP1), a key host factor that mediates viral entry [33,34]. (−)-Anisomelic acid inhibited binding of the viral spike protein with NRP1 in a dose-dependent manner (EC_50_ = 26.7 μM; Fig. 3A–C). This parallels the inhibitory effect of (−)-anisomelic acid on binding of the viral spike protein to the NRP1-positive (NRP1+) Huh7 cell line. This highlights the potential role of NRP1 in the viral entry process.

We evaluated the effects of (−)-anisomelic acid on the activity of enzymes that are possibly involved in the proteolytic processing of the viral spike protein [35,36]. We found that (−)-anisomelic acid potently inhibits the surface protease TMPRSS2 and lysosomal proteases cathepsin L and B with IC_50_ values of 6.51, 3.69 and 4.23 μM, respectively (Fig. 3D–F). These results indicate that (−)-anisomelic acid inhibits the entry process by blocking both receptor binding and proteolytic processing. Given that (−)-anisomelic acid inhibits the activities of the cysteine proteases cathepsin L and B, it probably also inhibits the SARS-CoV-2 main protease (3CL pro), which is the pivotal viral cysteine protease for viral replication [37]. To test this, we determined the inhibitory effect of (−)-anisomelic acid on 3CL pro; the IC_50_ value is 9.77 μM (Fig. 3G). We further carried out molecular modeling to analyse the binding modes of (−)-anisomelic acid with the potential targets. The docking results indicated that (−)-anisomelic acid favorably docked into the ligand binding site of NRP1 and the active sites of TMPRSS2, cathepsin L/B and main protease (Supplementary Fig. S5). These results show that (−)-anisomelic acid inhibits SARS-CoV-2 infection through multiple targets. Multi-targeting or polypharmacology is not uncommon for natural products, which have been used for thousands of years in traditional medicine [38,39]. In particular, multi-targeting [40] has advantages over single targeting, e.g. better efficacy, reduced toxicity and, more importantly, the ability to prevent the development of resistant viral strains and to enable the treatment of viral coinfections, e.g. in combination therapies for HIV and hepatitis C virus viral infections [41,42].

Most patients with severe COVID-19 have shown significantly increased serum levels of inflammatory cytokines such as IP10, IL6 and TNF-α, which are closely associated with lung injury and disease severity. This cytokine storm is induced by recognition of the viral double-strand RNA via host pattern recognition receptors such as RIG-I-like receptors or toll-like receptors [43]. To determine whether (−)-anisomelic acid ameliorates cytokines release syndrome, human THP-1 cells were transfected with poly (I : C), which is often used to stimulate antiviral signaling pathways [44]. Poly (I : C) transfection significantly induced expression of multiple cytokines, i.e. IFN-β, IP10, IL-1β, TNF-α, IL-6 and CCL2, whereas (−)-anisomelic acid modestly inhibited IFN-β expression and significantly suppressed the expression of IP10, IL-1β, TNF-α, IL-6 and CCL2, which are the hallmark cytokines of severe COVID-19 (Fig. 3H). These results suggest that (−)-anisomelic acid not only directly blocks SARS-CoV-2 infection, but also potentially attenuates the excessive host immune response that induces cytokine release syndrome.

**Figure 4.**
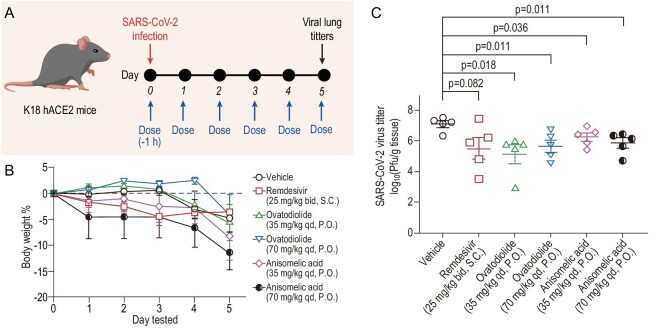
Reduction by (−)-anisomelic acid and (+)-ovatodiolide of SARS-CoV-2 infection in transgenic mice expressing human ACE2 *in vivo*. (A) Overview of *in vivo* study design. Mice were dosed at Day 0 with vehicle, remdesivir (25 mg/kg, bid, S.C.), (+)-ovatodiolide (35 or 70 mg/kg, qd. P.O.) or (−)-anisomelic acid (35 or 70 mg/kg, qd, P.O.) and treatment continued until Day 5. Mice were intranasally infected with SARS-CoV-2 1 h after the first dose at Day 0. Mice were sacrificed for analysis 4 h after administration of last dose. (B) Daily bodyweights of mice (*n* = 5 per group). Data are mean ± SD. (C) Virus loads in lung tissue of SARS-CoV-2-infected hACE2 transgenic mice (*n* = 5 per group) were titrated by using a plaque assay. Data are mean ± SEM. Two-tailed unpaired Student's *t*-test comparison with vehicle group. S.C., subcutaneous administration; P.O., oral administration.

Lastly, we evaluated the effect of (−)-anisomelic acid on SARS-CoV-2 infection *in vivo*. As shown in Fig. 4A, K18-hACE2 mice were infected with SARS-CoV-2 intranasally 1 h after administration of the first treatment dose [vehicle, remdesivir 25 mg/kg bid S.C., (−)-anisomelic acid 35 and 70 mg/kg qd P.O. and (+)-ovatodiolide 35 and 70 mg/kg qd P.O.]. The entire treatment lasted until Day 5. Four hours after administration of the last dose on Day 5, the mice were sacrificed and the viral loads in their lung tissues were determined. Figure 4B shows that there were no significant differences in body weight loss between the control group (vehicle) and all treated groups, indicating that the acute toxicity might not be a major concern [45] in either (−)-anisomelic acid or (+)-ovatodiolide, which was further supported by recent results reported on the toxicity study of (+)-ovatodiolide [46]. In terms of their antiviral effect, the viral titers in both (−)-anisomelic acid and (+)-ovatodiolide were lower than that of the control group, with reductions of 0.84–1.95 log units in viral lung titers (*P* < 0.05) (Fig. 4C). Remdesivir also reduced viral titters (1.61 log units) at the tested dose, albeit not significantly (*P* = 0.082). Taken together, these results show that both (−)-anisomelic acid and (+)-ovatodiolide effectively inhibit SARS-CoV-2 replication *in vivo*.

## CONCLUSION

In summary, the rapid global spread of SARS-CoV-2 highlights the urgent need for coronavirus therapeutics. We have shown that (−)-anisomelic acid (**1**) and (+)-ovatodiolide (**2**) can inhibit SARS-CoV-2 at similar levels to those achieved with remdesivir. Their antiviral effects were determined by replication in a CPE assay and in a K18-hACE2 mouse model of SARS-CoV-2 via oral administration. To accelerate future drug development, we have established an enantioselective synthesis of (−)-anisomelic acid (**1**) via a semisynthesis from naturally enriched (+)-costunolide (**5**) in five steps; the total yield was 27%. This provides opportunities for large-scale, cost-effective production. Given that *Anisomeles* herbal species have traditionally been used for the treatment of various plagues in China over hundreds of years, (−)-anisomelic acid (**1**) could be a promising orally bioavailable lead compound for the development of therapeutics against COVID-19.

## METHODS

Experimental procedures, characterization of new compounds and all other data supporting the findings are available in the Supplementary data.

## DATA AVAILABILITY

Refined single-crystal X-ray crystallographic data for the structure of (−)-anisomelic acid (**1**) are available free of charge from the Cambridge Crystallographic Data Centre under deposition number 2116979.

## Supplementary Material

nwac176_Supplemental_File
