# Supplementary material for: Identification and semisynthesis of (−)-anisomelic acid as oral agent against SARS-CoV-2 in mice
Source: Natl Sci Rev. 2022 Aug 26;9(11):nwac176. doi: 10.1093/nsr/nwac176 (PMC9798891; doi:10.1093/nsr/nwac176)
Supplement: nwac176_Supplemental_File [file nwac176_supplemental_file.pdf]

Supplementary Materials

Identification and Semi-synthesis of (−)-Anisomelic Acid as an Oral Antiviral Agent against SARS-CoV-2 in Mice

Hai-Xin Yu, Nan Zheng, Chi-Tai Yeh, Chien-Ming Lee, Qi Zhang, Wen-Lv Zheng, Qing Chang, Yuan-He Li, Yu-Jun Li, Gui-Zhen Wu, Jun-Min Quan, Lin-Qi Zhang, Yew-Min Tzeng, Zhen Yang

This PDF file includes:

In vitro Antiviral Activity Assays

In vivo Antiviral Activity Assays

Materials and Methods

Figs. S1 to S3

Spectral Data

Single Crystal XRD Data

References

In vitro *Antiviral Activity Assays* *1*

Cells and viruses 1

***In vitro* Cell-based antiviral activity assays** 1

Cellular viability assays 2

Real-time PCR 2

Indirect ELISA inhibition of (−)-anisomelic acid on the host cell human neuropilin-1 (NRP1) receptor 3

TMPRSS2 activity inhibition 3

Cathepsin L and B protease activity inhibition 3

***In vitro* inhibition of (−)-anisomelic acid on the host cell human neuropilin-1 (NRP1) receptor** 4

***In vitro* inhibition of (−)-anisomelic acid on SARS-CoV-2 main protease (M pro)** 4

Molecular Modelling *5*

In vivo *Antiviral Activity Assays* *5*

***In vivo* SARS-CoV-2 challenge** 5

Viral load and viral titre detections 6

Statistical analysis 6

Supporting Figures *7*

Figure S1. Detailed route to (−)-anisomelic acid (SI-1) 7

Figure S2. Detailed route to SI-6 7

Figure S3. Detailed route to SI-7 7

Figure S4. Use specific inhibitors as a positive control for SARS-CoV 2 enzymatic activity inhibition. (A) Campstat/TMPRSS2. (B) E-64/cathepsin L. (C) E-64/cathepsin B. (D) Tannin acid/3CL pro. 8

Figure S5. Predicted binding modes of AA and Ova with potential targets 8

Procedure and Characteristic Data for Chemical Synthesis *9*

General Information 9

Isolation of (−)-anisomelic acid and (+)-ovatodiolide 9

Experimental Procedure and Compound Characterization **9**

SI-4 .... **10**

SI-6 .... **10**

SI-15 .... **11**

SI-7 .... **12**

SI-8 and SI-9 .... **13**

SI-3 .... **14**

SI-10 and SI-11 .... **15**

(−)-Anisomelic acid (SI-1) **17**

Comparison of NMR data for natural and synthetic (−)-anisomelic acid **18**

Comparison of ^{1}H NMR data for (−)-anisomelic acid **18**

Comparison of ^{13}C NMR data for (−)-anisomelic acid **19**

Comparison of the spectra for natural and synthetic (−)-anisomelic acid **21**

NMR Spectral Data ***22***

^{1}H & ^{13}C NMR Spectra for SI-4 .... **22**

^{1}H & ^{13}C NMR Spectra for SI-6 .... **23**

^{1}H & ^{13}C NMR Spectra for SI-15 .... **24**

^{1}H & ^{13}C NMR Spectra for SI-7 .... **25**

^{1}H & ^{13}C NMR Spectra for SI-8 .... **26**

^{1}H & ^{13}C NMR Spectra for SI-8 .... **27**

^{1}H & ^{13}C NMR Spectra for SI-3 .... **27**

^{1}H & ^{13}C NMR Spectra for SI-10 .... **29**

^{1}H & ^{13}C NMR Spectra for SI-11 .... **29**

^{1}H & ^{13}C NMR Spectra for (−)-anisomelic acid (SI-1) **30**

Single Crystal XRD Data ***32***

References ***37***

In vitro Antiviral Activity Assays

Cells and viruses

Vero E6 cells (ATCC-1586) were maintained in Dulbecco's modified eagle medium (DMEM, Gibco) supplemented with 10% heat-inactivated fetal bovine serum (FBS, Gibco), 100 U/mL penicillin and streptomycin at 37 °C in a humidified atmosphere of 5% CO_2. THP1 cells were purchased from SIBS (Shanghai, China), and cultured in RPMI1640 supplemented with 10% FBS and 0.1% Normocin (InvivoGen, Catalogue #ant-ar-1) at 37 °C with 5% CO_2. human airway epithelial Calu-3 cells were obtained from Procell (Wuhan, China, #CL-0054), and cultured in PM150410 supplemented with 20% FBS and 1% P/S (PB180120). SARS-CoV-2 pseudotyped viruses were generated by co-transfection of human immunodeficiency virus backbones expressing firefly luciferase (pNL43R-E-luciferase) and pcDNA3.1 (Invitrogen) expression vectors encoding the respective S proteins into 293T cells (ATCC). Viral supernatants were collected 48 h later. Viral titers were measured as luciferase activity in relative light units (Bright-Glo Luciferase Assay Vector System, Promega Biosciences). The SARS-CoV-2/F13/environment/2020/Wuhan (F13) was isolated from an environmental sample collected in the Huanan Seafood Market in Wuhan. The SARS-CoV-2 Delta (B.1.617.2, GDPCC 2.00096) variants were isolated from patients with COVID-19 admitted in the Guangzhou Eighth People's Hospital by Center for Disease Control and Prevention of Guangdong Province. Live SARS-CoV-2 infection experiments were performed in the BSL-3 laboratory at the China CDC.

In vitro Cell-based antiviral activity assays

HIV pseudotyped with spike protein (S protein) of SARS-CoV-2 were generated according to a published protocol. Inhibition assays were performed by incubating pseudoviruses with serial dilutions of (−)-anisomelic acid and (+)-ovatodiolide at 37 °C for 1 h, respectively. HeLa-hACE2 cells (1×10^5 per well) were added in duplicate to the virus-drug mixture. Half-maximal inhibitory concentrations (IC_{50}) of (−)-anisomelic acid and (+)-ovatodiolide were determined by luciferase activity 48 h after exposure to virus-drug mixture using GraphPad Prism 6 (GraphPad Software Inc.). To evaluate the antivirus activity against the live SARS-CoV-2 virus, the cytopathic effect (CPE) assay was performed in a BSL-3 laboratory of national Institute for Viral Disease Control

and Prevention, China CDC. the prototype (F13 Strain), viruses were used in the assays. The tested drugs were serially diluted and mixed with an equal volume of 100 $TCID_{50}$ live SARS-CoV-2 virus. After incubation at 37 °C for 2 h, the mixed solution was added into the well of the plate containing Vero E6 cells with density of 2×10^5 per mL, which was then cultured at 37 °C for 4 days. Both cell and virus controls were also set up as comparison. Subsequently, the inhibition of virus infection to the cells was observed, and the molecule titer against the live SARS-CoV-2 virus was measured as the reciprocal of the serum dilution for 50% neutralization of viral infection. To evaluate the activities of compounds against viral infection on human cell lines, Calu-3 cells were seeded at 1×10^5 cells per well in 24-well plates. Cells were allowed to adhere for 24 h and then infected at an MOI of 0.05 with SARS-CoV-2 for 1 h at 37 °C. Then the viral inoculum was removed, and the cells were washed two times with prewarmed phosphate-buffered saline (PBS). Medium containing dilutions of compounds was added. At 48 hpi, supernatants or cells were harvested for qRT-PCR analysis as previously reported.[1] The dose−response curves were plotted from viral RNA copies versus the drug concentrations using GraphPad Prism 6 software.

Cellular viability assays

To evaluate the cytotoxic effect of drugs on host cells, Vero E6 cells were suspended in growth medium in 96-well plates at a density of 5,000 cells/well for 24 h and then treated with drugs at serial concentrations for 24 h. Cell viability was tested by using Cell Counting Kit-8 (CCK-8, APExBio, K1018).

Real-time PCR

For determination of mRNA expression levels, 8×10^5 THP1 cells was transfected with poly (I:C) in 1 mL Opti-MEM (GBICO, 31985-070) in 6-well plates in the absence of or in the presence of anisomelic acid (10 μM). Total RNA was isolated with total RNA extraction reagent (Takara, 9108), and was reverse transcribed using the Hifair 1st first strand cDNA synthesis supermix (Yeasen Biotech, 11141ES60). IFNβ, IP10, TNFa, IL-6, CCL2 and GAPDH mRNA levels were measured with the Hieff qPCR SYBR Green Master Mix kit (Yeasen Biotech, 11201ES08) on the CFX96 Real-Time PCR system (Biorad, Unite states). Samples were carried out in triplicate and normalized to GAPDH levels.

Indirect ELISA inhibition of (−)-anisomelic acid on the host cell human neuropilin-1 (NRP1) receptor

For the indirect ELISA, 100 ng of each protein was coated onto MaxiSORP plates (Nunc) using 100 mM carbonate buffer and blocked with Gelatin Blocking Buffer. SARS-CoV-2 spike S1 monoclonal antibody (Cat.No: E-AB-V1005), according to the manufacturer's manual, conjugated HRP or Alexa Fluor® 488 as biomarkers were tested at a dilution of 1:200. For the competitive ELISA, MaxiSORP plates (Nunc) were coated with 5 μg/mL of recombinant Human Neuropilin-1 (Elabscience) in bicarbonate buffer overnight at 4 °C. Wells were blocked using Gelatin Blocking Buffer for 2 h at 37 °C and recombinant SARS-CoV-2 spike S1 with 20 μg/well and incubated for 2 h at 37 °C. Following extensive washing, SARS-CoV-2 spike S1 monoclonal antibody at 1:1000 dilution was added and incubated for 1h at 37 °C. The chromogenic reaction was quantified following the addition of TMB substrate (Invitrogen) and stop solution (KPL SeraCare). The absorbance of the samples was measured at 450 nm and the background at 570 nm.

TMPRSS2 activity inhibition

TMPRSS2 fluorescence detection kit (BPS BIOSCIENCE, 78083). According to the manufacturer's manual, the inhibitory activity was measured by kinetic analysis. The sample containing TMPRSS2 was reacted with a fluorescent substrate in a mixture, and the measured fluorescence (λ^{ex} = 383 nm, λ^{em} = 455 nm) was read. Using camostat as a positive control for protease inhibitors. The single reaction dose of TMPRSS2 was 150 ng/group, and 10 μM camostat was used as a positive control for inhibition in a 50 μL reaction system. We tested various concentrations of Anisomelic acid (0.5, 1, 2.5, 5, 10, 20, and 30 μM) to examine the effect of Anisomelic acid on the inhibition of TMPRSS2 activity.

Cathepsin L and B protease activity inhibition

Cathepsin L and B fluorescence detection kit (BPS BIOSCIENCE, 79591 and 79590). According to the manufacturer's manual, the inhibitory activity was measured by kinetic analysis. The sample containing Cathepsin L and B was reacted with a fluorescent substrate in a mixture, and the measured fluorescence (λ^{ex} = 360 nm, λ^{em} = 460 nm) was read. Using E64 as a positive control for protease inhibitors. The single reaction dose of Cathepsin L was 0.4 ng/group, and E64 was used as a positive control for inhibition in a 50 μL reaction system. We tested various concentrations of

(−)-anisomelic acid (0.5, 1, 2.5, 5, 10, 20, and 30 μM) to examine the effect of (−)-anisomelic acid on the inhibition of Cathepsin L and B protease activity.

***In vitro* inhibition of (−)-anisomelic acid on the host cell human neuropilin-1 (NRP1) receptor**

The human hepatoma cell lines (Huh 7) were purchased from American Tissue Culture Collection. The cells were maintained under conditions recommended by ATCC. Adherent cells were grown in RPMI-1640 medium supplemented with 10% fetal bovine serum, streptomycin (100 μg/mL), and penicillin (100 IU/mL) as a monolayer culture in a humidified 5% CO_2 atmosphere at 37 °C and were subcultured every 48 – 72 h. Regarding cellular immunofluorescence staining, Huh 7 cells were washed with phosphate buffered saline (PBS). Then the cells were exposed to 5% paraformaldehyde for 20 minutes and 10 minutes, the cell membrane was dehydrated and permeated at -20 °C, and then immersed in 1% bovine serum albumin (BSA)/PBS for 30 minutes for treatment. Huh7 cells were incubated overnight at 4 °C in a primary polyclonal antibody ACE2 for receptor blocking. In the control group, different concentrations of anisomelic acid (respectively 0, 5, 15, 30, 60 μM) and a fixed concentration of new coronavirus S1 recombinant protein 20 μg were added to test the effect of (−)-anisomelic acid on blocking the S1 binding to host NRP1 receptors. Subsequent detection of fluorescent signal uses a monoclonal antibody that can detect S1 and is conjugated with Alexa Fluor 488 (Invitrogen, Thermo Fisher Scientific Corp., Carlsbad, CA, USA) for staining, and stained with 4',6-diamidino-2-Nuclear staining with phenylindole (DAPI, Millipore). Read the slide under the microscope and use Image J for image analysis.

***In vitro* inhibition of (−)-anisomelic acid on SARS-CoV-2 main protease (M pro)**

The viral main proteinase (M pro, also called 3CL pro), which controls the activities of the coronavirus replication complex. It functions as a cysteine protease engaging in the proteolytic cleavage of the viral precursor polyprotein to a series of functional proteins required for coronavirus replication and is considered as an appealing target for designing anti-SARS agents. Recombinant 2019-nCoV 3C-like Proteinase Protein (Elabscience bio lnc) expression from E. Coli. The catalytic activity of M pro was measured by continuous kinetic assays, using an identical fluorogenic substrate Dabcyl-KTSAVLQSGFRKME-Edans (synthetic). Fluorogenic peptide is used as the substrate and the enhanced fluorescence due to cleavage of this substrate catalyzed by the protease is monitored at 538 nm with an excitation wavelength of 355 nm using a fluorescence plate reader.

Sensitive internally quenched fluorogenic (FRET) substrate for SARS main protease. The experiments were performed in a 100-μL reaction system with a buffer consisting of 50 mM Tris · HCl (pH 7.3), 1 mM (ethylenedinitrilo)tetraacetic acid. To measure the EC_{50} of a compound, 500 nM of enzyme, 20 μM of substrate, and the Anisomelic acid at seven different concentrations were added into different wells. The compound was dissolved and diluted in dimethyl sulfoxide to the desired concentrations. One microliter of diluted compound was added into 50 μL of solution containing 1 μM M pro, and then solutions were incubated at room temperature for 10 min. The reaction was initiated by adding 50 μL of substrate. Fluorescence intensity was monitored once every 45 s. Initial reaction velocities were calculated by fitting the linear portion of the curves (within the first 5 min of the progress curves) to a straight line using the program SoftMax Pro and were converted to enzyme activity (substrate cleaved)/second. The inhibitory effect of anisomelic acid on the main protease activity. We examined the effect that (−)-anisomelic acid has on the 3CL-protease activity by testing various concentrations of (−)-anisomelic acid (0.5, 1, 2.5, 5, 10, 20, and 30 μM). We found that (−)-anisomelic acid significantly inhibits 3CL-protease activity in a dose-dependent manner.

Molecular Modelling

The docking template structures of potential targets were derived from the crystal structure deposited in PDB protein data bank (PDB code: 2ORZ for NRP1; 7MEQ for TMPRSS2; 3OF9 for cathepsin L; 3K9M for cathepsin B; 7BUY for Mpro). Docking was done using the latest version AutoDock Vina.[2] The illustrated structures were made by PyMOL.[3]

In vivo Antiviral Activity Assays

In vivo SARS-CoV-2 challenge

Human ACE2 transgenic mice (K18-hACE2) were generated by microinjection of the mouse ACE2 promoter driving the human ACE2 coding sequence into the pronuclei of fertilized ova from ICR mice, and then human ACE2 integrated was identified by PCR as previously described. hACE2 transgenic ICR mice were challenged with 1×10^5 plaque forming unit (PFU) SARS-CoV-2 via intranasal inoculation. Mice body weights were monitored daily over a 6-day time course.

Subcutaneous administration of remdesivir (25 mg/kg, bid)[4] and oral gavage administration (35 mg/kg/day and 70 mg/kg/day, respectively) with (−)-anisomelic acid and (+)-ovatodiolide one day prior to infection and continued dosing for 5 days, respectively. Mice were dissected at 5 dpi to collect different tissues to evaluate virus replication and histopathological changes. All protocols were approved by the SRI IACUC (Institutional Animal Care and Use Committee) and all procedures used in this study complied with the guidelines and policies of the Animal Care and Use Committee of SRI with an approved animal use protocol of 20003.

Viral load and viral titer detections

Total RNA was extracted from tissues homogenates of organs using the RNeasy Mini Kit (Qiagen), and reverse transcription was performed using the PrimerScript RT Reagent Kit (TaKaRa) following the manufacturers' instructions. RT-qPCR reactions were performed using the PowerUp SYBG Green Master Mix Kit (Applied Biosystems). The primer sequences used for RT-qPCR are targeted against the envelope (E) gene of SARS-CoV-2 and are as follows: forward: 5'-TCGTTTCGGAAGAGACAGGT-3', reverse: 5'-GCGCAGTAAGGATGGCTAGT-3'. Samples were carried out in triplicate and normalized to GAPDH levels, and viral loads were expressed as log10-transformed numbers of genome equivalent copies per ml of sample. Virus titers in the lungs were determined using focus FFA assay as previously described. Briefly, Vero E6 cells were seeded in 96-well plates one day before infection. Lung homogenates were serially diluted and used to inoculate Vero E6 cells at 37 °C for 1 h. The supernatants were removed before adding with virus medium containing 1% CMC (carboxymethyl cellulose) then incubated at 37 °C with 5% CO_2 for 24 h. Cells were fixed with 10% paraformaldehyde and permeabilized with 0.1% Triton X-100. Cells were then incubated with a rabbit anti-SARS-CoV-2 nucleocapsid protein polyclonal antibody, followed by an HRP-labeled goat anti-rabbit secondary antibody. The foci were visualized by TrueBlue Peroxidase Substrate (KPL), and counted with an ELISPOT reader (Cellular Technology). The virus titers were calculated and expressed as fluorescent focus units (PFU) per gram tissue.

Statistical analysis

All data were analyzed with GraphPad Prism 8.0 software. Statistically significant differences were determined using unpaired *t*-tests, Student's *t*-tests, Welch's *t*-tests or Mann-Whitney U-tests, as applicable and according to test requirements. A two-sided P value < 0.05 was considered statistically significant. *$P < 0.05$, **$P < 0.01$, ***$P < 0.001$.

Supporting Figures

Figure S1. Detailed route to (−)-anisomelic acid (SI-1)

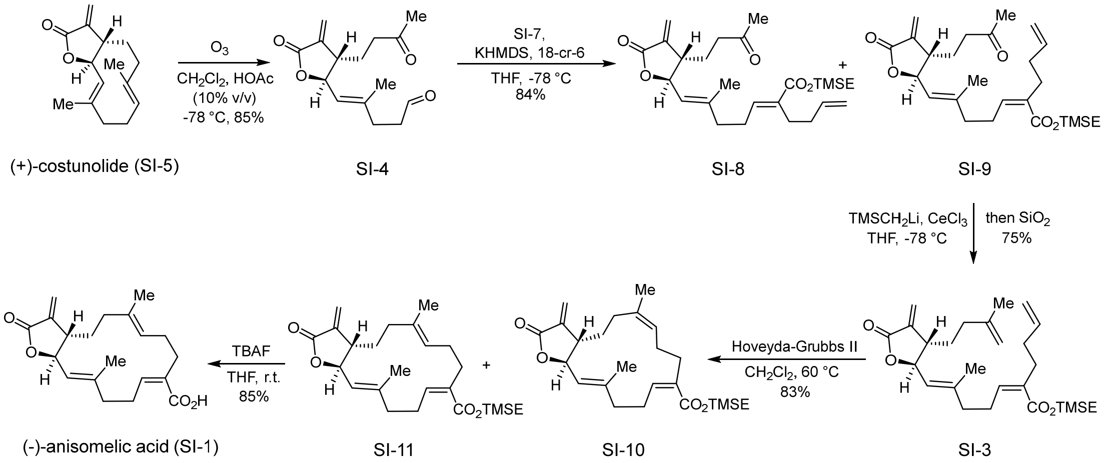

Figure S2. Detailed route to SI-6

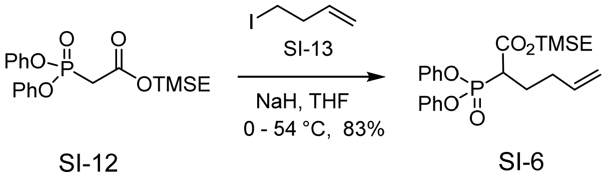

Figure S3. Detailed route to SI-7

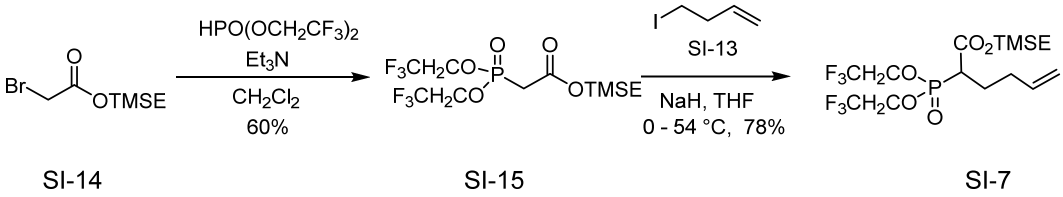

Figure S4. Use specific inhibitors as a positive control for SARS-CoV-2 enzymatic activity inhibition. (A) Campstat. (B) E-64/cathepsin L. (C) E-64/cathepsin B. (D) Tannin acid/3CL pro.

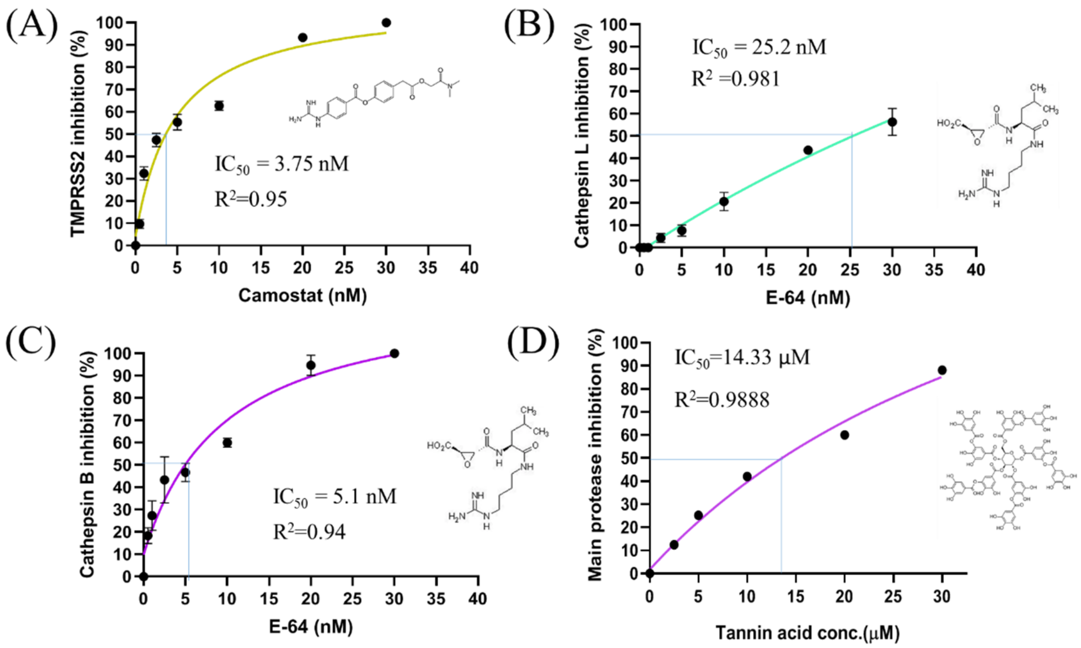

Figure S5. Predicted binding modes of AA and Ova with potential targets.

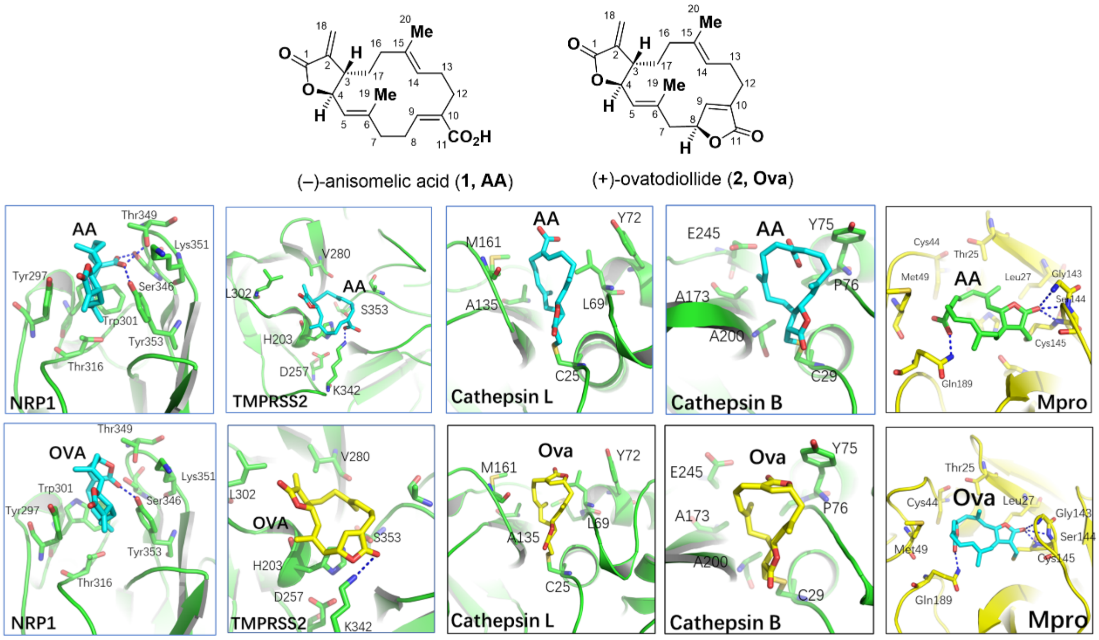

Procedure and Characteristic Data for Chemical Synthesis

General Information

Unless otherwise noted, all reactions were carried out under a nitrogen atmosphere under anhydrous conditions and all reagents were purchased from commercial suppliers without further purification. Anhydrous tetrahydrofuran (THF) was distilled from sodium-benzophenone. Dichloromethane (DCM) was distilled from calcium hydride. Reactions were monitored by thin-layer chromatography (TLC) carried out on 0.25 mm Tsingdao silica gel plates (GF-254) using UV light as visualizing agent and an ethanolic solution of phosphomolybdic acid (PMA) and cerium sulfate, and heat as developing agents. Tsingdao silica gel (60, particle size 0.040-0.063 mm) was used for flash column chromatography. NMR spectra were recorded on a Brüker Advance 400 (^{1}H-400 MHz, ^{13}C-100 MHz), Brüker Advance 500 (^{1}H-500 MHz, ^{13}C-125 MHz); All spectral data were acquired at 295 K. The following abbreviations were used to explain the multiplicities: s = singlet, d = doublet, t = triplet, q = quartet, m = multiplet; IR spectra were recorded on an IRPrestige-21 FTIR spectrometer. High resolution mass spectrometric (HRMS) data were obtained using Brüker Apex IV RTMS.

Isolation of (−)-anisomelic acid and (+)-ovatodiolide

The materials used in our research were isolated prepared from the dried leaves. Accordingly, *A. indica* (4.0 kg) were steeped in EtOAc (3.0 L) at room temperature for 4 days, and the resultant mixture was filtrated off, and the filtrate was concentrated under vacuum, and the residue was purified via flash column chromatography on silica gel (EtOAc: Hexanes = 1:4) to give (−)-anisomelic acid as a yellowish solid (0.83 g, mp: 151.1-153.3 °C) and (+)-ovatodiolide as a yellowish solid (3.32 g, mp: 146-147 °C), respectively, after recrystallization from a mixed solvent of EtOAc and hexanes (4:6 = v/v). The ^{1}H and ^{13}C NMR spectra and specific rotation of the isolated compounds were in agreement with those reported in the literature[5]

Experimental Procedure and Compound Characterization

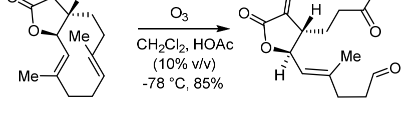

(+)-costunolide (SI-5) SI-4

SI-4

(+)-Costunolide **SI-5** (800 mg, 3.54 mmol, 1.0 eq.) was dissolved in 10% v/v AcOH containing DCM (25 mL) and the resultant mixture was cooled to -78 °C. Freshly prepared ozone was carefully bubbled in and the reaction process was monitored by TLC until complete consumption of (+)-Costunolide. Dimethyl sulfide (1.0 mL) was added and the mixture was allowed to warm up to room temperature. The residue was slowly diluted with a saturated solution of $NaHCO_3$ until bubbling ceased, and the mixture was then extracted with DCM (3 × 20 mL). The combined organic layers were washed with brine and dried over sodium sulfate. The solvent was removed under vacuum, and the residue was purified by a flash column chromatography on silica gel (petroleum ether : EtOAc = 8:1 to 4:1) to give **SI-4** (773 mg, 85% yield) as colorless oil. (*Note: **SI-4** was unstable under high vacuum and long-term storage and should be used as soon as possible*).

$[\alpha]_D^{22}$ = +26.1 (c = 0.12, $CHCl_3$);

^{1}H NMR: (400 MHz, $CDCl_3$) δ 9.75 (s, 1H), 6.24 (d, *J* = 2.8 Hz, 1H), 5.57 (d, *J* = 2.5 Hz, 1H), 5.17 (d, *J* = 8.3 Hz, 1H), 4.75 (dd, *J* = 8.9, 6.1 Hz, 1H), 2.72 (dt, *J* = 8.3, 5.8 Hz, 1H), 2.57 – 2.37 (m, 5H), 2.19 – 2.06 (m, 4H), 1.95 (dt, *J* = 13.7, 7.4 Hz, 1H), 1.85 – 1.75 (m, 4H);

^{13}C NMR: (101 MHz, $CDCl_3$) δ 207.29, 201.26, 170.00, 142.50, 138.76, 123.25, 122.02, 79.36, 45.06, 41.53, 39.69, 31.39, 30.07, 25.66, 17.17;

IR ν_{max} (film): 2949, 2730, 1726, 1684, 1450, 1389, 1250, 1189, 737 cm^{-1};

HRMS (ESI) m/z: $C_{15}H_{20}NaO_4$ $[M+Na]^+$ calcd: 287.1254, found: 287.1248.

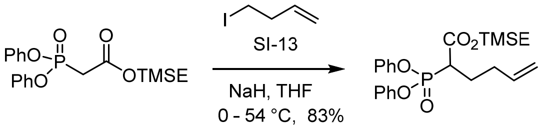

SI-12 SI-6

SI-6

Reagent **SI-12** was prepared as described previously.[6] To a stirred solution of **SI-12** (5.0 g, 12.7 mmol, 1.0 eq.) in THF (25 mL) was added NaH (60% dispersion in mineral oil, 720 mg, 1.4 eq.) in portions at 0 °C. The mixture was allowed to stir at 0 °C for another 1 h after bubbling ceased. Freshly prepared 4-iodobut-1-ene (3.9 g, 21.6 mmol, 1.7 eq.) dissolved in THF (10 mL) was added

to the mixture in a dropwise manner. The mixture was then heated to 54 °C for 48 h. Upon completion as monitored by TLC, the reaction was carefully quenched by adding saturated NH_4Cl solution, and the mixture was then extracted with EtOAc (3 × 25 mL). The combined organic extracts were washed with brine (50 mL), and dried over sodium sulfate. The solvent was concentrated under vacuum, and the residue was purified by a flash chromatography on silica gel (petroleum ether : EtOAc = 20:1) to give **SI-6** (4.7 g, 83% yield) as a yellowish oil.

^{1}H NMR: (500 MHz, $CDCl_3$) δ 7.31 (dd, *J* = 14.9, 7.4 Hz, 4H), 7.18 (dd, *J* = 13.6, 6.4 Hz, 6H), 5.77 (ddt, *J* = 12.6, 10.2, 6.2 Hz, 1H), 5.06 (dd, *J* = 13.7, 7.1 Hz, 2H), 4.31 – 4.21 (m, 2H), 3.31 (ddd, *J* = 23.1, 10.5, 2.8 Hz, 1H), 2.39 – 2.08 (m, 4H), 1.06 – 0.95 (m, 2H), 0.04 (d, *J* = 0.6 Hz, 9H);

^{13}C NMR: (126 MHz, CDCl3) δ 168.34, 136.49, 129.85, 125.46, 120.66, 116.68, 115.50, 64.38, 45.88, 44.82, 32.34, 32.21, 26.27, 26.23, 17.50, -1.45;

IR ν_{max} (film): 3442, 2920, 1696, 1415, 1257, 1230, 861cm^{-1};

HRMS (ESI) m/z: $C_{23}H_{31}NaO_5PSi$ [M+Na]$^+$ calcd: 496.1571, found: 496.1571.

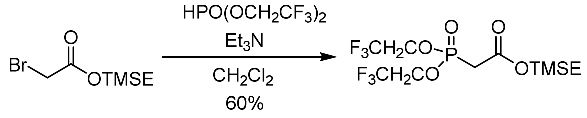

SI-14 SI-15

SI-15

To an ice cold solution of Bis(2,2,2-trifluoroethyl)phosphite (4.03 g, 16.4 mmol, 1 eq.) in DCM (17 mL) was added 2-trimethylsilylethyl bromoacetate **SI-14** (3.92 g, 16.4 mmol, 1eq.), followed by Et_3N (1.66 g, 2.28 mL, 16.4 mmol, 1 eq.). After stirring for 15 min at 0 °C, the reaction mixture was warmed up to room temperature and stirring was continued for an additional 48 h. The reaction mixture was quenched by addition of saturated $NaHCO_3$ aqueous solution (20 mL). The aqueous layer was extracted with DCM (3 × 15 mL), the organic layers were dried over sodium sulfate and concentrated under vacuum. The resulting oil was purified flash column chromatography on silica gel (petroleum ether : EtOAc = 10:1) to give **SI-15** as a yellow oil (3.97 g, 60%).

^{1}H NMR: (400 MHz, $CDCl_3$) δ 4.54 – 4.35 (m, 4H), 4.31 – 4.09 (m, 2H), 3.15 (s, 1H), 3.10 (s, 1H), 1.08 – 0.86 (m, 2H), 0.03 (s, 9H).

^{13}C NMR: (100 MHz, CDCl3) δ 164.81 (d, *J* = 4.2 Hz), 122.42 (qd, *J* = 277.5, 8.5 Hz), 64.80, 62.55 (qd, *J* = 38.1, 5.5 Hz), 34.09 (d, *J* = 144.6 Hz), 17.22, -1.67.

IR ν_{max} (film): 2958, 2905, 1742, 1734, 1714, 1301, 1265, 1255, 1100, 1071, 963, 860, 840 cm^{-1}

HRMS (ESI) m/z: $C_{11}H_{19}F_6NaO_5PSi$ [M+Na]$^+$: calcd: 427.0536; found: 427.0536.

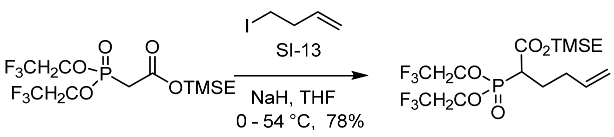

SI-15 SI-7

SI-7

To a stirred solution of **SI-15** (3.97 g, 9.84 mmol, 1.0 eq.) in THF (25 mL) was added NaH (60% dispersion in mineral oil, 551 mg, 13.78 mmol, 1.4 eq.) in portions at 0 °C. The mixture was allowed to stir at 0 °C for another 1 h after bubbling ceased. Freshly prepared 4-iodobut-1-ene (3.02 g, 16.72 mmol, 1.7 eq.) dissolved in THF (10 mL) was added to the mixture in a dropwise manner. The mixture was then heated to 54 °C for 48 h. Upon completion as monitored by TLC, the reaction was carefully quenched by adding saturated NH_4Cl solution, and the mixture was then extracted with EtOAc (3 × 25 mL). The combined organic extracts were washed with brine (50 mL), and dried over sodium sulfate. The solvent was concentrated under vacuum, and the residue was purified by flash chromatography on silica gel (petroleum ether : EtOAc = 10:1) to give **SI-6** (3.52 g, 78% yield) as a yellowish oil.

^{1}H NMR: (400 MHz, $CDCl_3$) δ 5.79 – 5.65 (m, 1H), 5.10 – 5.05 (m, 1H), 5.03 (d, *J* = 1.2 Hz, 1H), 4.49 – 4.29 (m, 4H), 4.29 – 4.20 (m, 2H), 3.12 (ddd, *J* = 21.9, 9.7, 4.0 Hz, 1H), 2.32 – 1.82 (m, 4H), 1.05 – 0.99 (m, 2H).

^{13}C NMR: (100 MHz, CDCl3) δ 168.10 (d, *J* = 3.7 Hz), 135.99, 122.45 (qd, *J* = 278.8, 8.1 Hz), 116.81, 64.67, 64.23 – 59.50 (m), 44.76 (d, *J* = 136.6 Hz), 31.77 (d, *J* = 15.0 Hz), 26.08 (d, *J* = 5.2 Hz), 17.32, -1.62.

IR ν_{max} (film): 2958, 2902, 1742, 1732, 1729, 1418, 1299, 1172, 1071, 963, 861 cm^{-1}

HRMS (ESI) m/z: $C_{15}H_{25}F_6NaO_5PSi$ [M+Na]$^+$: calcd: 481.1005; found: 481.1008.

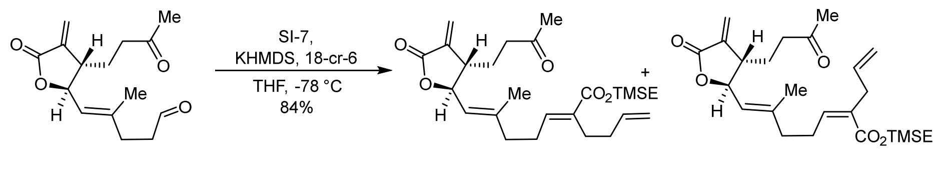

SI-8 and SI-9

To a solution of 18-crown-6 (845 mg, 3.2 mmol) in THF (10 mL) was added potassium bis(trimethylsilyl)amide (2.80 mL, 1.0 M in THF, 2.80 mmol) at -78 °C, and the resulting mixture was stirred at the same temperature for 10 min. To this mixture was added a solution of reagent **SI-7** (1.37 g, 3.0 mmol) in THF (2.0 mL) at -78 °C, and resulting mixture was then stirred at the same temperature for another 10 min. Followed by addition of a solution of ketoaldehyde **SI-4** (625 mg, 2.0 mmol) in THF (2.0 mL) at -78 °C, and the resulting mixture was stirred for 1 h. The reaction mixture was quenched with a saturated solution of NH_4Cl (10 mL) at - 78 °C, and the formed mixture was then extracted with EtOAc (3 × 10 mL). The combined organic layers were washed with brine (15 mL), and dried over sodium sulfate. The solvent was removed under vacuum, and the residue was purified by a flash column chromatography on silica gel (petroleum ether/ethyl acetate = 2:1) to give **SI-8** (111 mg, 12% yield) as a yellowish oil and **SI-9** (662 mg, 72% yield) as a yellowish oil.

SI-8

$[\alpha]_D^{22}$ = +31.8 (c = 0.17, $CHCl_3$);

^{1}H NMR: (500 MHz, $CDCl_3$) δ 6.70 (t, *J* = 7.3 Hz, 1H), 6.29 (d, *J* = 2.9 Hz, 1H), 5.80 (ddt, *J* = 17.0, 10.1, 6.8 Hz, 1H), 5.59 (d, *J* = 2.5 Hz, 1H), 5.22 (dd, *J* = 9.0, 1.2 Hz, 1H), 5.05 – 4.93 (m, 2H), 4.80 (dd, *J* = 9.0, 5.9 Hz, 1H), 4.26 – 4.18 (m, 2H), 2.83 – 2.69 (m, 1H), 2.57 – 2.43 (m, 2H), 2.42 – 2.35 (m, 2H), 2.31 (dd, *J* = 15.2, 7.5 Hz, 2H), 2.21 – 2.12 (m, 7H), 2.03 – 1.92 (m, 1H), 1.86 (tt, *J* = 14.4, 7.2 Hz, 1H), 1.80 (d, *J* = 1.2 Hz, 3H), 1.02 (ddd, *J* = 10.5, 7.2, 3.8 Hz, 2H), 0.05 (s, 9H);

^{13}C NMR: (126 MHz, $CDCl_3$) δ 207.06, 169.98, 167.78, 143.20, 141.06, 138.82, 137.91, 132.59, 123.21, 121.97, 115.12, 79.41, 62.76, 45.13, 39.67, 38.47, 33.37, 30.06, 26.66, 26.46, 25.77, 17.42, 17.06, -1.44;

IR ν_{max} (film): 3440, 3310, 2926, 2375, 1262, 1250, 1019, 1011, 861, 837, 799 cm^{-1};

HRMS (ESI) m/z: $C_{26}H_{40}NaO_5Si$ $[M+Na]^+$: calcd: 483.2537; found: 483.2539.

SI-9

$[\alpha]_D^{21}$ = +24.0 (c = 0.1, $CHCl_3$);

^{1}H NMR: (500 MHz, $CDCl_3$) δ 6.28 (d, *J* = 2.9 Hz, 1H), 5.82 – 5.72 (m, 2H), 5.58 (d, *J* = 2.5 Hz, 1H), 5.20 (dd, *J* = 9.1, 1.2 Hz, 1H), 5.03 – 4.93 (m, 2H), 4.80 (dd, *J* = 9.1, 5.9 Hz, 1H), 4.25 – 4.19 (m, 2H), 2.78 – 2.71 (m, 1H), 2.58 (dd, *J* = 15.2, 7.4 Hz, 2H), 2.50 (t, *J* = 7.6 Hz, 2H), 2.35 – 2.30 (m, 2H), 2.20 – 2.13 (m, 7H), 1.98 (ddd, *J* = 14.0, 7.4, 6.0 Hz, 1H), 1.84 (td, *J* = 14.4, 7.7 Hz, 1H), 1.78 (d, *J* = 1.3 Hz, 3H), 1.06 – 1.01 (m, 2H), 0.05 (s, 9H);

^{13}C NMR: (126 MHz, $CDCl_3$) δ 207.06, 167.97, 143.67, 140.51, 138.92, 137.80, 132.35, 122.89, 121.85, 115.09, 79.54, 62.46, 45.14, 39.66, 39.06, 34.00, 33.36, 30.08, 27.43, 25.76, 17.53, 16.99, -1.47 ppm;

IR ν_{max} (film): 3310. 2926, 2375, 1507, 1262, 1019, 1011, 851, 837, 799 cm^{-1};

HRMS (ESI) m/z: $C_{26}H_{40}NaO_5Si$ $[M+Na]^+$: calcd: 483.2537; found: 483.2537.

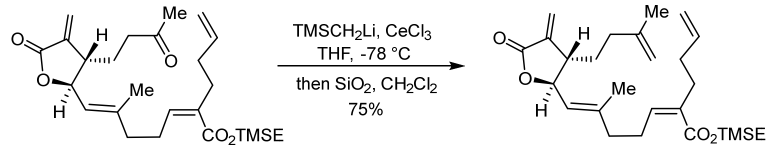

SI-9 SI-3

SI-3

To an anhydrous cerium (III) chloride (493 mg, 2.0 mmol; dried at 150 °C under vacuum for 3 h) was added THF (4.0 mL) at 0 °C, and the resultant mixture was stirred at room temperature for 24 h. To this mixture was added (trimethylsilyl) methyllithium (1.5mL, 1.0 M in pentane, 1.5 mmol) at -78 °C, and the resultant mixture was stirred at the same temperature for 1 h. After addition of a solution of compound **SI-9** (460 mg, 1.0 mmol) in THF (2.0 mL) to the above prepared reaction mixture at -78 °C, the resultant mixture was stirred for 1 h, and quenched with a 10% v/v AcOH containing water (10 mL), and the mixture was extracted with EtOAc (3×10 mL). The combined organic layers were washed with brine (10 mL), dried over sodium sulfate, and concentrated under vacuum. The residue was dissolved in DCM (5.0 mL) and silica gel (2.30 g) was added. The suspension was stirred for 12 hours, filtered and washed with ethyl acetate (3 × 10 mL). The solvent

was concentrated under vacuum and the residue was purified by flash chromatography on silica gel (petroleum ether : EtOAc = 20:1 to 10:1) to give compound **SI-3** (341 mg, 75 % yield) as a colorless oil.

$[\alpha]_D^{23}$ = +32.7 (c = 0.35, $CHCl_3$);

^{1}H NMR: (500 MHz, $CDCl_3$) δ 6.26 (d, *J* = 2.8 Hz, 1H), 5.82 – 5.70 (m, 2H), 5.57 (d, *J* = 2.5 Hz, 1H), 5.22 (dd, *J* = 9.1, 0.8 Hz, 1H), 5.04 – 4.92 (m, 2H), 4.84 (dd, *J* = 9.1, 5.7 Hz, 1H), 4.76 (s, 1H), 4.68 (s, 1H), 4.25 – 4.19 (m, 2H), 2.74 – 2.67 (m, 1H), 2.57 (dd, *J* = 15.1, 7.4 Hz, 2H), 2.34 – 2.27 (m, 2H), 2.19 – 2.12 (m, 4H), 2.05 (t, *J* = 7.9 Hz, 2H), 1.86 – 1.75 (m, 4H), 1.75 – 1.62 (m, 4H), 1.10 – 0.96 (m, 2H), 0.05 (s, 9H);

^{13}C NMR: (126 MHz, $CDCl_3$) δ 170.31, 167.99, 144.37, 142.94, 140.58, 139.42, 137.81, 132.27, 123.19, 121.50, 115.06, 110.96, 79.85, 62.44, 45.48, 39.05, 34.37, 34.02, 33.38, 30.77, 27.36, 22.41, 17.52, 16.92, -1.47;

IR ν_{max} (film): 2845, 2410, 1825, 1260, 1176, 1132, 1114, 1012, 934, 857, 835, 797 cm^{-1};

HRMS (ESI) m/z: $C_{27}H_{42}NaO_4Si$ $[M+Na]^+$: calcd: 481.2745; found: 481.2743.

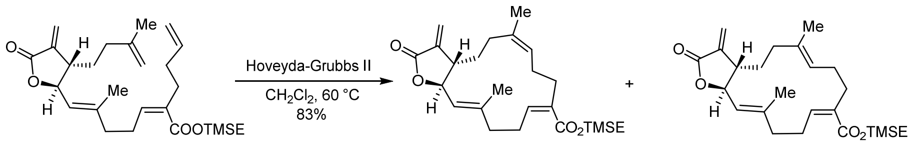

SI-3 SI-10 SI-11

SI-10 and SI-11

To a stirred solution of **4** (100 mg, 0.22 mmol, 1.0 eq.) in DCM (1000 mL) was added Hoveyda-Grubbs II catalyst (6.8 mg, 0.01 mmol, 0.05 eq.). The resultant mixture was degassed multiple times with argon and heated to reflux (60 °C) for 48 h. The reaction was worked up by removal of the solvent of the reaction mixture under vacuum, and the residue was purified by a flash chromatography on silica gel (petroleum ether : EtOAc = 50:1 to 20:1 to 4:1) to give **SI-11** (65 mg, 69% yield) and **SI-10** (13 mg, 14% yield) as colorless oils.

SI-10

$[\alpha]_D^{24}$ = -12.1 (c = 0.1, $CHCl_3$);

^{1}H NMR: (400 MHz, $CDCl_3$) δ 6.23 (d, *J* = 3.2 Hz, 1H), 5.75 (dd, *J* = 10.1, 4.2 Hz, 1H), 5.55 (d, *J* = 2.9 Hz, 1H), 5.30 (d, *J* = 8.9 Hz, 1H), 5.21 (t, *J* = 7.9 Hz, 1H), 4.73 (t, *J* = 8.6 Hz, 1H), 4.22 – 4.15 (m, 2H), 3.14 – 2.99 (m, 1H), 2.69 (dd, *J* = 8.1, 3.3 Hz, 1H), 2.59 – 2.50 (m, 1H), 2.38 – 2.19 (m, 5H), 2.13 – 2.03 (m, 3H), 1.98 – 1.92 (m, 1H), 1.81 (s, 4H), 1.68 (s, 3H), 1.03 (dd, *J* = 9.9, 7.6 Hz, 2H), 0.06 (s, 9H);

^{13}C NMR: (101 MHz, CDCl3) δ 170.50, 168.19, 145.53, 141.93, 140.27, 135.90, 134.14, 125.10, 123.71, 120.34, 80.01, 62.49, 47.17, 39.17, 35.26, 30.28, 29.93, 29.71, 25.84, 23.00, 17.73, 16.43, -1.38;

IR ν_{max} (film): 2970, 2888, 2834, 1718, 1709, 1530, 1377, 1245, 1132, 926, 847 **cm^{-1};**

HRMS (ESI) m/z: $C_{25}H_{38}NaO_4Si$ $[M+Na]^+$: calcd: 453.2432; found: 453.2430.

SI-11

$[\alpha]_D^{24}$ = -48.7 (c = 0.24, $CHCl_3$);

^{1}H NMR: (400 MHz, $CDCl_3$) δ 6.24 (d, *J* = 2.6 Hz, 1H), 5.64 (t, *J* = 6.6 Hz, 1H), 5.57 (d, *J* = 2.3 Hz, 1H), 5.21 – 5.15 (m, 1H), 5.05 – 4.96 (m, 1H), 4.88 (dd, *J* = 9.7, 4.0 Hz, 1H), 4.23 (ddd, *J* = 8.0, 5.0, 1.3 Hz, 2H), 2.86 – 2.73 (m, 1H), 2.71 – 2.56 (m, 2H), 2.47 (dd, *J* = 13.1, 6.6 Hz, 1H), 2.32 – 2.02 (m, 7H), 1.78 (d, *J* = 1.0 Hz, 3H), 1.76 – 1.67 (m, 2H), 1.59 (s, 3H), 1.07 – 0.99 (m, 2H), 0.05 (s, 9H);

^{13}C NMR: (101 MHz, $CDCl_3$) δ 170.58, 168.13, 142.21, 141.22, 140.76, 132.19, 131.00, 125.61, 124.18, 121.66, 79.17, 62.52, 43.04, 38.49, 36.18, 34.71, 32.30, 25.71, 25.13, 17.67, 16.59, 15.76, -1.40.

IR ν_{max} (film): 2910, 2891, 2884, 1729, 1718, 1570, 1384, 1262, 1122, 936, 857 **cm^{-1};**

HRMS (ESI) m/z: $C_{25}H_{38}NaO_4Si$ $[M+Na]^+$: calcd: 453.2432; found: 453.2430.

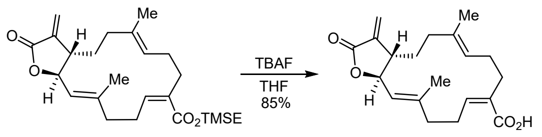

SI-11 (-)-anisomelic acid (SI-1)

(−)-Anisomelic acid (SI-1)

To a stirred solution of **SI-11** (50 mg, 0.12 mmol, 1.0 eq.) in THF (5.0 mL) was added dropwise TBAF (1.0 M in THF, 0.17 mL, 1.4 eq.) and the resultant mixture was allowed to stir at room temperature until complete consumption of **SI-11**. Then saturated NH_4Cl solution was added to quench the reaction and pH was adjusted to 5.0 using 0.5 N HCl. The mixture was then extracted with EtOAc (3 × 10 mL). The combined organic extracts were washed with brine (100 mL) and dried over sodium sulfate. The solvent was concentrated under vacuum and the residue was purified by flash chromatography on silica gel (petroleum ether : EtOAc = 4:1 to 2:1) to give **(−)-anisomelic acid (SI-1)** (34 mg, 85 % yield) as a white crystal.

$[\alpha]_D^{22}$ = -18.3 (c = 0.8, $CHCl_3$);

^{1}H NMR: (500 MHz, $CDCl_3$) δ 6.25 (d, *J* = 2.6 Hz, 1H), 5.88 (t, *J* = 6.5 Hz, 1H), 5.59 (d, *J* = 2.3 Hz, 1H), 5.18 (d, *J* = 9.6 Hz, 1H), 4.99 (d, *J* = 5.3 Hz, 1H), 4.88 (dd, *J* = 9.6, 4.2 Hz, 1H), 2.88 (ddd, *J* = 21.6, 14.2, 7.1 Hz, 1H), 2.77 – 2.64 (m, 2H), 2.50 (t, *J* = 13.4 Hz, 1H), 2.36 – 2.16 (m, 6H), 2.11 – 2.02 (m, 1H), 1.78 (d, *J* = 0.8 Hz, 3H), 1.76 – 1.63 (m, 2H), 1.60 (s, 3H).

^{13}C NMR: (126 MHz, $CDCl_3$) δ 173.11, 170.62, 146.93, 141.15, 140.66, 132.52, 129.68, 125.36, 124.36, 121.77, 79.16, 43.07, 38.46, 36.18, 34.44, 32.21, 26.16, 25.08, 16.64, 15.83;

IR ν_{max} (film): 2975, 2899, 2837, 1748, 1729, 1662, 1387, 1268, 1126, 946, 815 **cm^{-1};**

HRMS (ESI) m/z: $C_{20}H_{26}NaO_4$ $[M+Na]^+$: calcd: 353.1723; found: 353.1723.

Comparison of NMR data for natural and synthetic (−)-anisomelic acid

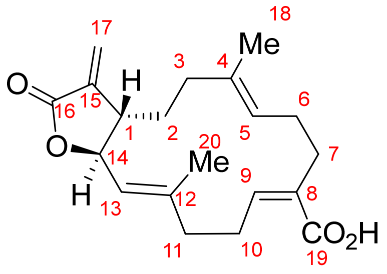

(-)-anisomelic acid (SI-1)

Comparison of ^{1}H NMR data for (−)-anisomelic acid

Position	Synthetic<br>500 MHz<br>δ H [ppm, mult, *J* (Hz)]	Natural[7]<br>200 MHz<br>δ H [ppm, mult, *J* (Hz)]	Natural[8]<br>400 MHz<br>δ H [ppm, mult, *J* (Hz)]
**1**	2.36 – 2.16 (m, 1 H)	–	2.38 – 2.15 (m, 1 H)
**2**	1.76 – 1.63 (m, 2 H)	–	1.76 – 1.63 (m, 2 H)
**3**	2.77 – 2.64 (m, 2 H)	–	2.80 – 2.61 (m, 2 H)
**4**	–	–	–
**5**	4.99 (d, *J* = 5.3, 1 H)	4.99 (m)	5.05 – 4.94 (m, 1 H)
**6**	2.36 – 2.16 (m, 2 H)	–	2.38 – 2.15 (m, 2 H)
**7**	2.36 – 2.16 (m, 2 H)	–	2.38 – 2.15 (m, 2 H)
**8**	–	–	–
**9**	5.88 (t, *J* = 6.5, 1 H)	5.87 (dd, *J* = 5.9, 6.8)	5.88 (t, *J* = 6.4, 1 H)
**10**	2.50 (t, *J* = 13.4, 1 H)<br>2.11 – 2.02 (m, 1 H)	–	2.51 (d, *J* = 11.9, 1 H)<br>2.07 (t, *J* = 11.8, 1 H)
**11**	2.88 (ddd, *J* = 21.6, 14.2, 7.1, 1H), 2.36 – 2.16 (m, 1 H)	–	2.97 – 2.81 (m, 1 H), 2.38 – 2.15 (m, 1 H)
**12**	–	–	–
**13**	5.18 (d, *J* = 9.6 Hz, 1 H)	5.18 (dd, J = 1.2, 9.8)	5.18 (d, *J* = 9.6, 1 H)
**14**	4.88 (dd, *J* = 9.6, 4.2, 1 H)	4.89 (dd, J = 4.3, 9.8)	4.88 (dd, *J* = 9.6, 4.1, 1 H)

**15**	–	–	–
**16**	–	–	–
**17**	6.25 (d, *J* = 2.6, 1 H),<br>5.59 (d, *J* = 2.3, 1 H)	6.25, 5.59, (d, *J* = 2.4)	6.25 (d, *J* = 2.4, 1 H),<br>5.59 (d, *J* = 1.9, 1 H)
**18**	1.60 (s, 3 H)	–	1.60 (s, 3 H)
**19**	–	–	–
**20**	1.78 (d, *J* = 0.8, 3 H)	1.78 (d, *J* = 1.2, 3 H)	1.78 (s, 3 H)

Comparison of ^{13}C NMR data for (−)-anisomelic acid

**Position**	**Synthetic**<br>**126 MHz**<br>δ C (ppm)	**Natural**[7]<br>**50.3 MHz**<br>δ C (ppm)	**Natural**[8]<br>**101 MHz**<br>δ C (ppm)
**1**	43.07	40.90	43.0
**2**	25.08	24.91	25.1
**3**	26.16	26.00	26.1
**4**	129.68	129.49	129.7
**5**	124.36	124.13	124.3
**6**	32.21	32.03	32.2
**7**	36.18	36.00	36.2
**8**	141.15	141.04	141.1
**9**	146.93	146.84	147.0
**10**	34.44	34.26	34.4
**11**	38.46	38.25	38.4
**12**	140.66	140.44	140.6
**13**	125.36	125.20	125.3
**14**	79.16	79.00	79.2
**15**	132.52	132.28	132.5
**16**	173.11	173.05	173.3
**17**	121.77	121.71	121.1

**18**	15.83	15.63	15.8
**19**	170.62	170.54	170.6
**20**	16.64	16.40	16.6

Comparison of the spectra for natural and synthetic (−)-anisomelic acid

Synthetic (−)-anisomelic acid

^{1}H NMR Spectrum

(500 MHz, $CDCl_3$)

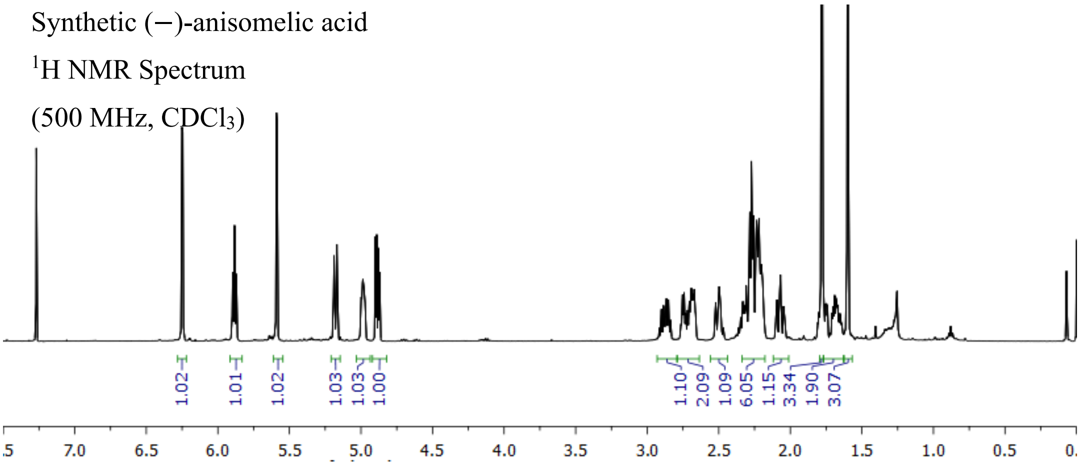

Natural (−)-anisomelic acid

^{1}H NMR Spectrum

(400 MHz, $CDCl_3$)

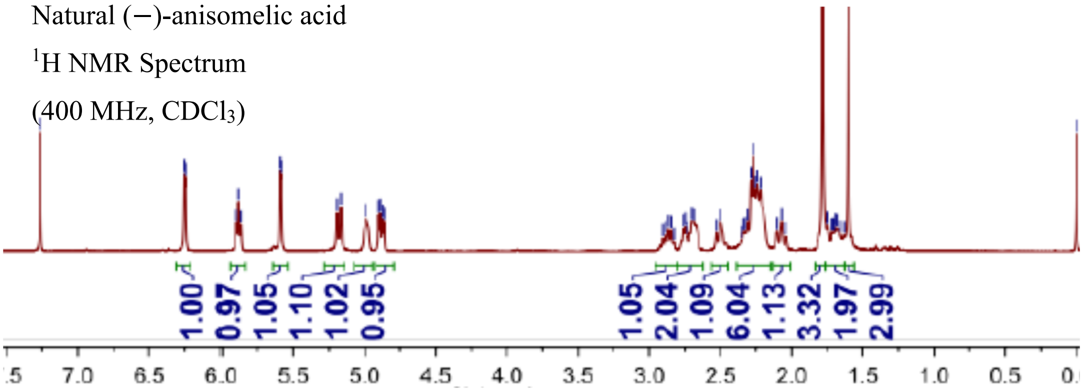

Synthetic (−)-anisomelic acid

^{13}C NMR Spectrum

(126 MHz, $CDCl_3$)

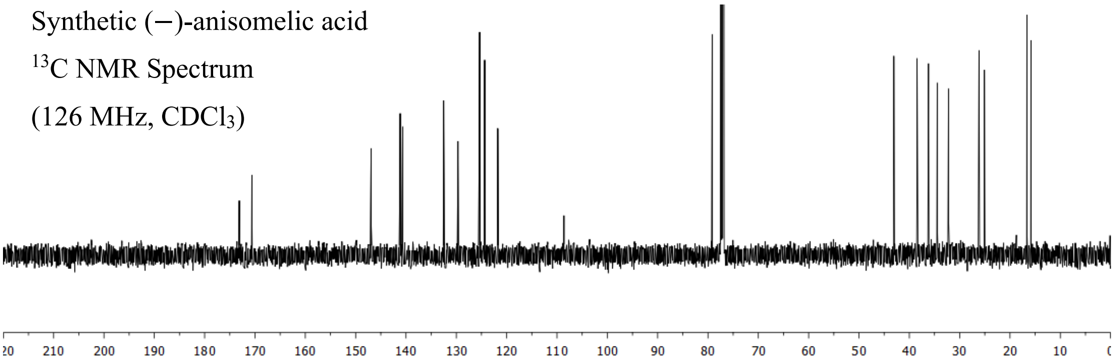

Natural (−)-anisomelic acid

^{13}C NMR Spectrum

(126 MHz, $CDCl_3$)

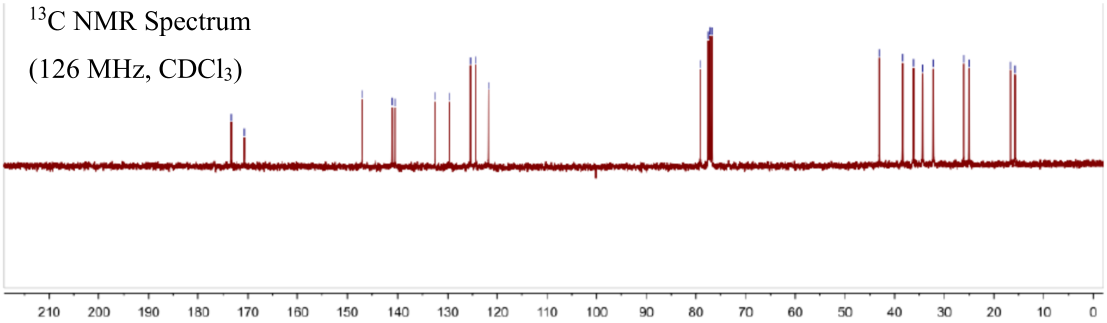

NMR Spectral Data

^{1}H & ^{13}C NMR Spectra for **SI-4**

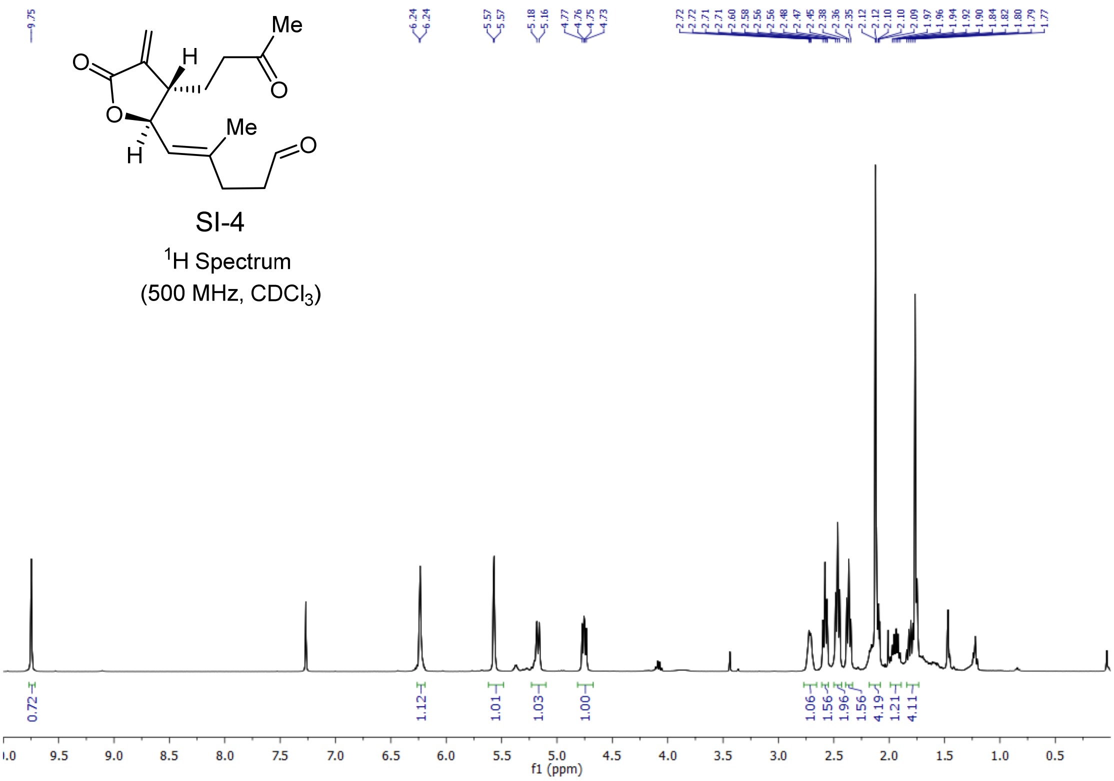

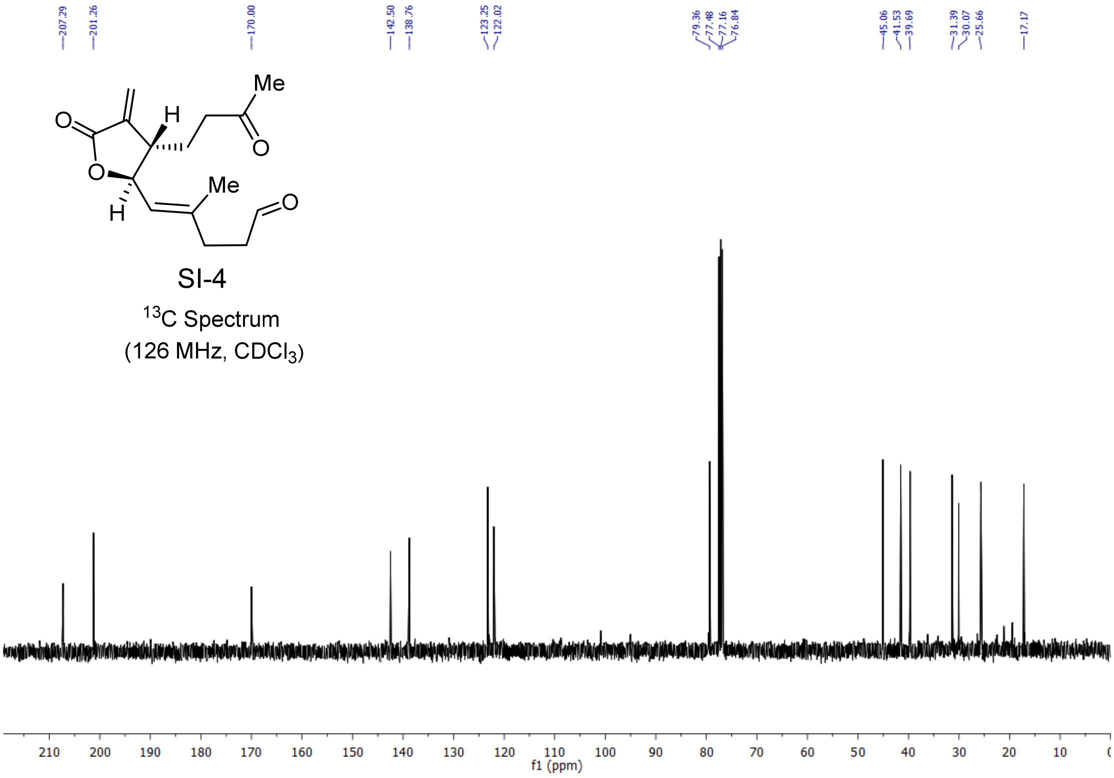

^{1}H & ^{13}C NMR Spectra for **SI-6**

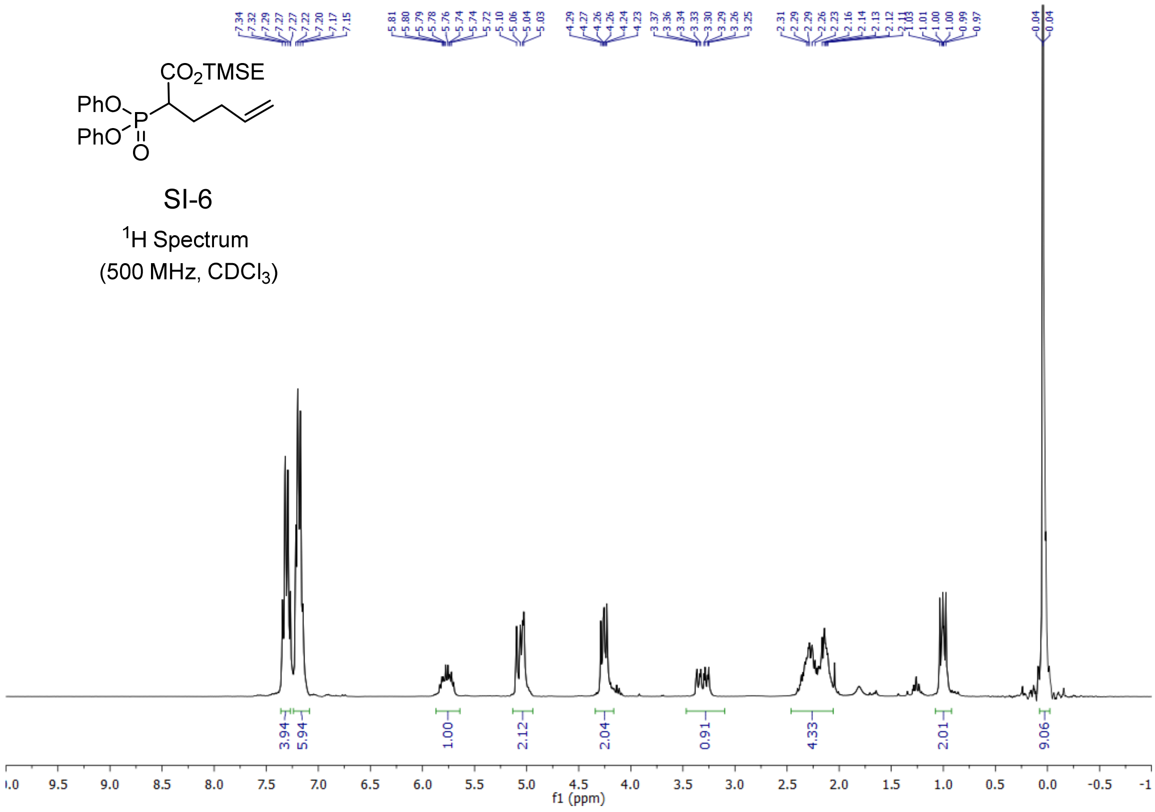

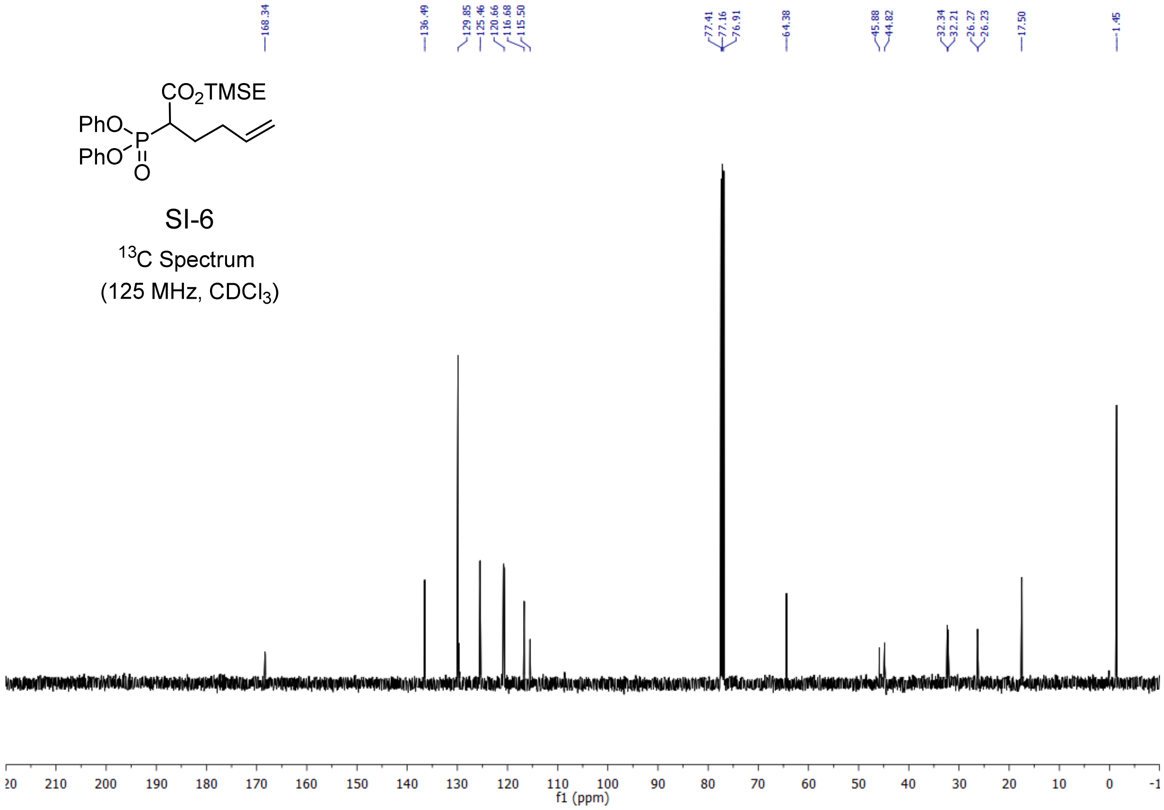

^1H & ^{13}C NMR Spectra for **SI-15**

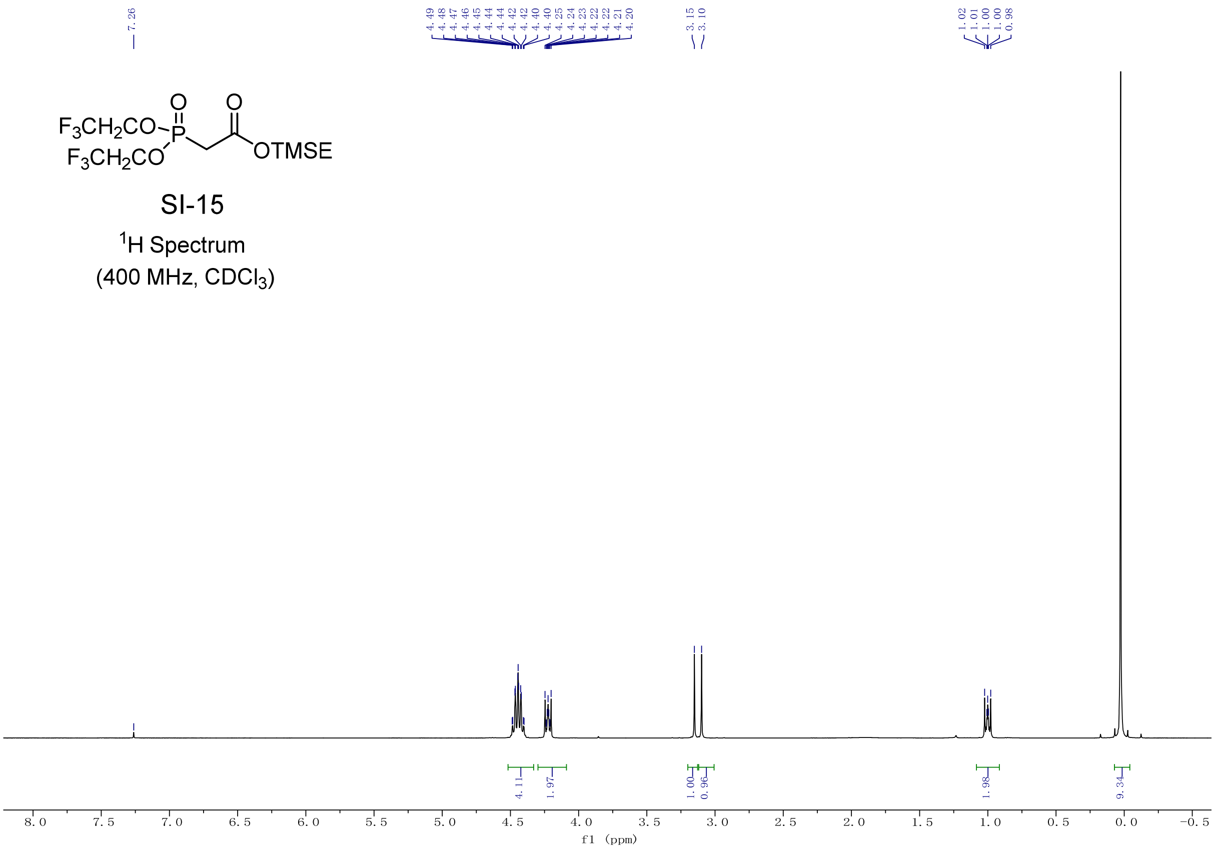

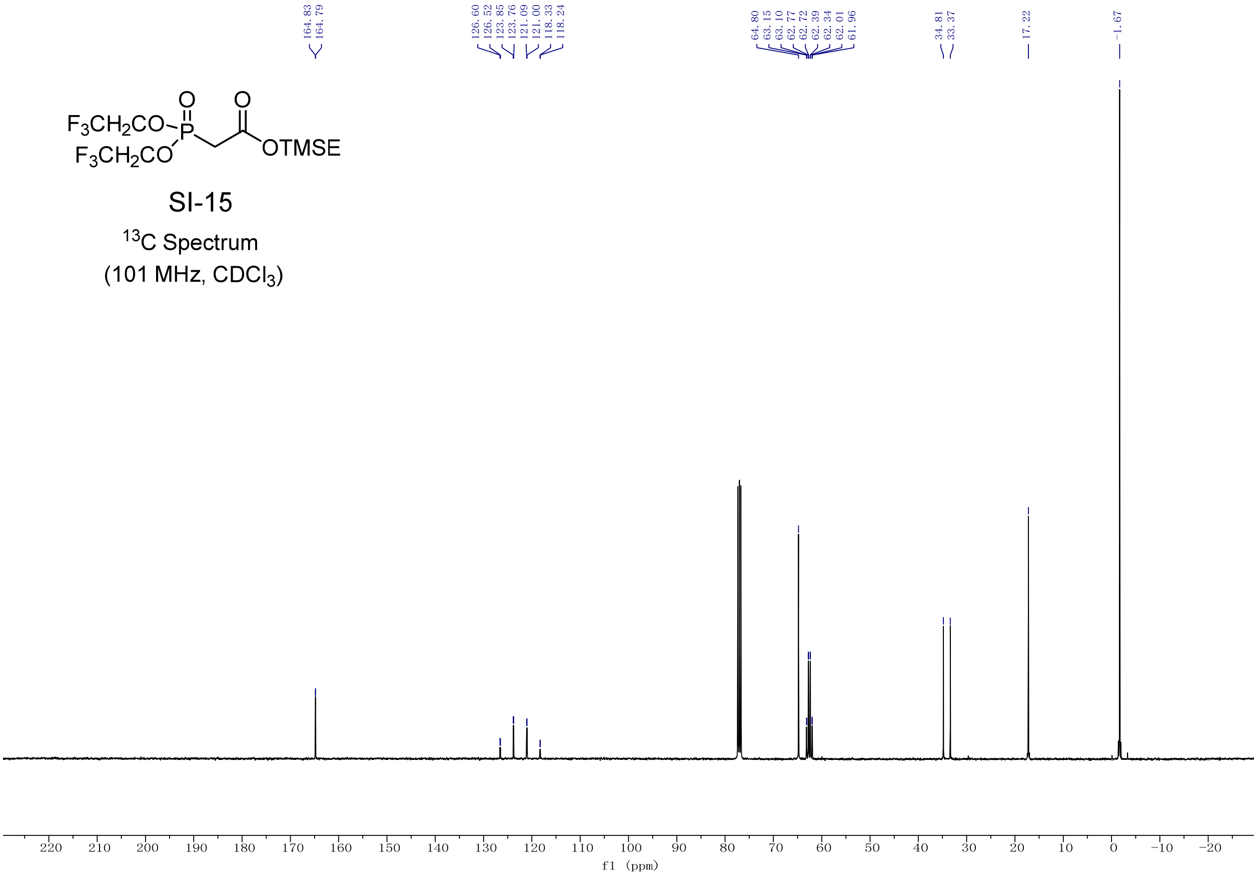

^{1}H & ^{13}C NMR Spectra for **SI-7**

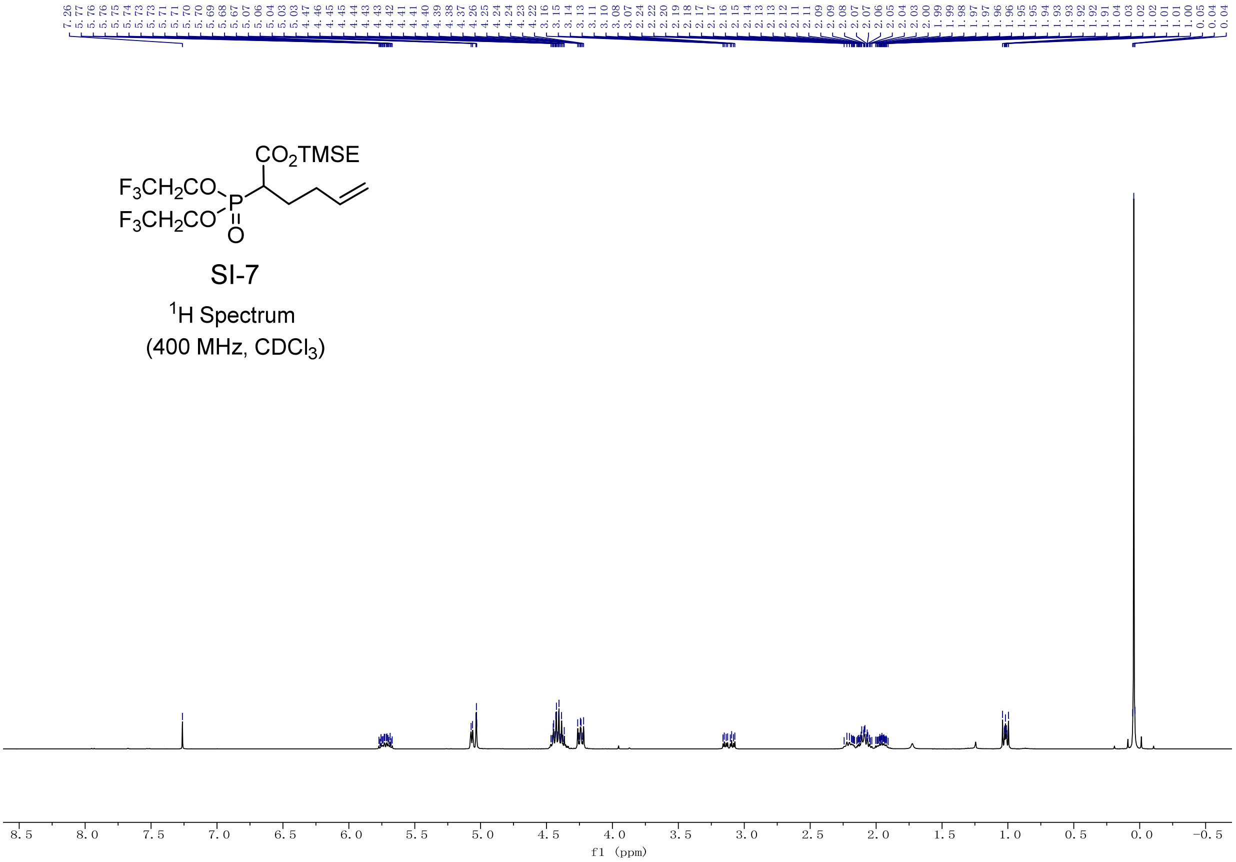

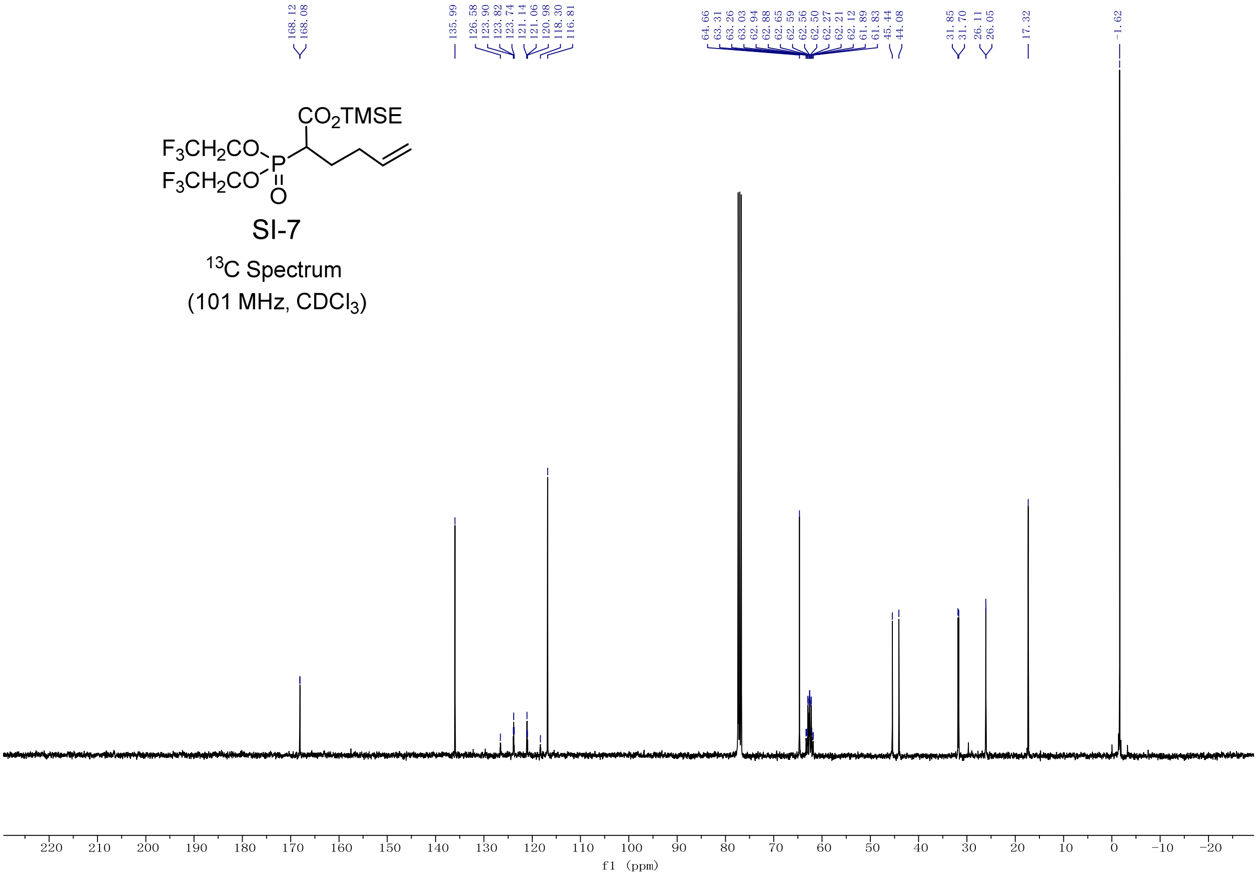

^{1}H & ^{13}C NMR Spectra for **SI-8**

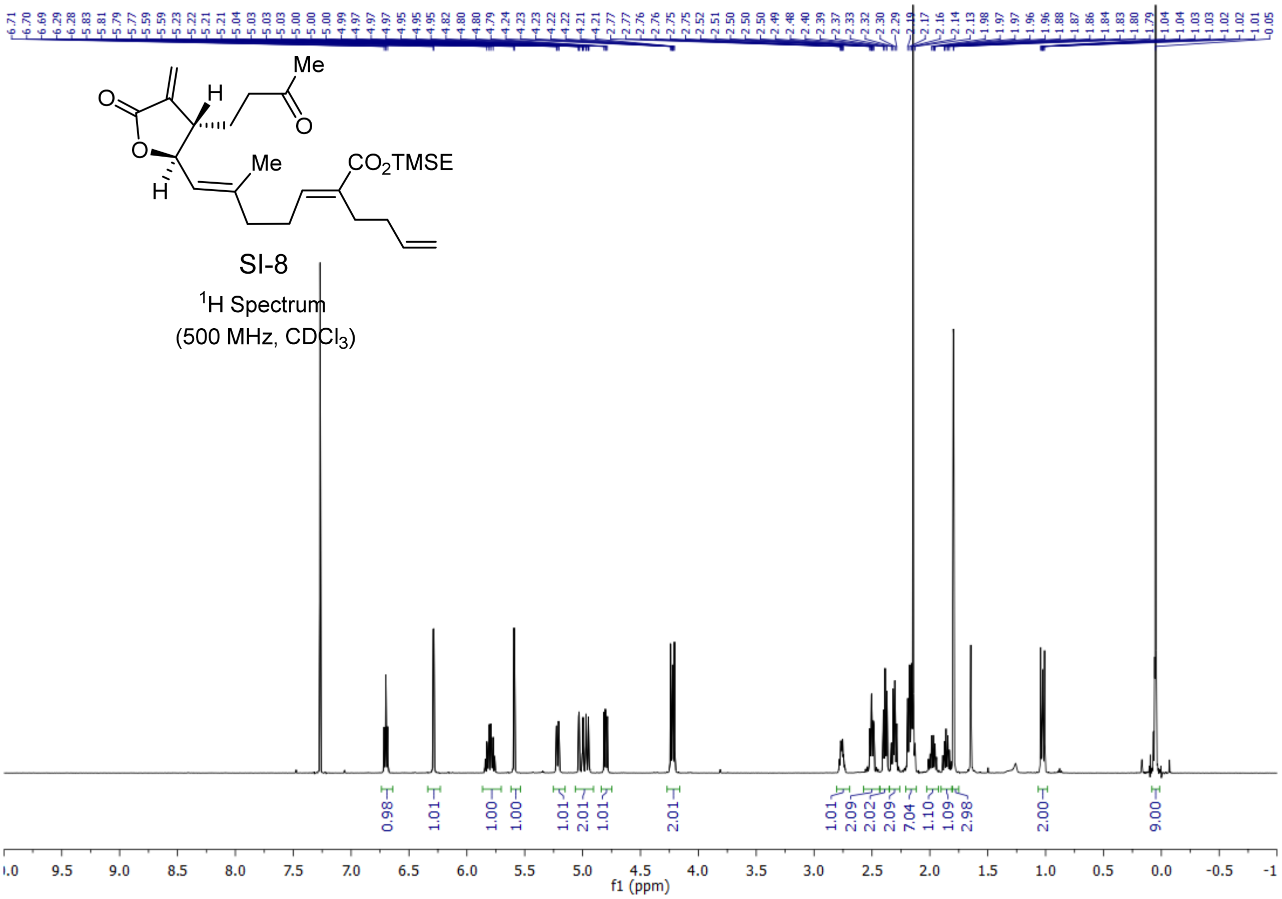

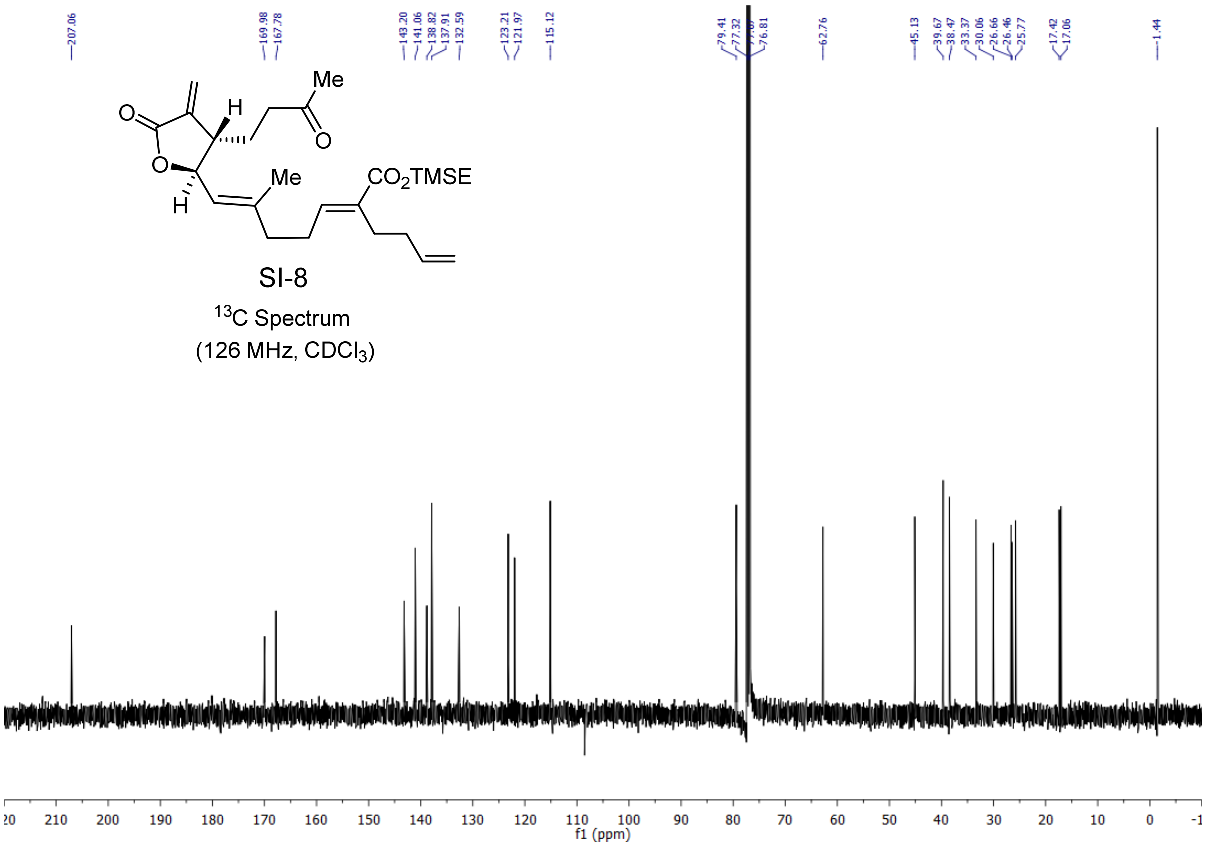

^{1}H & ^{13}C NMR Spectra for **SI-8**

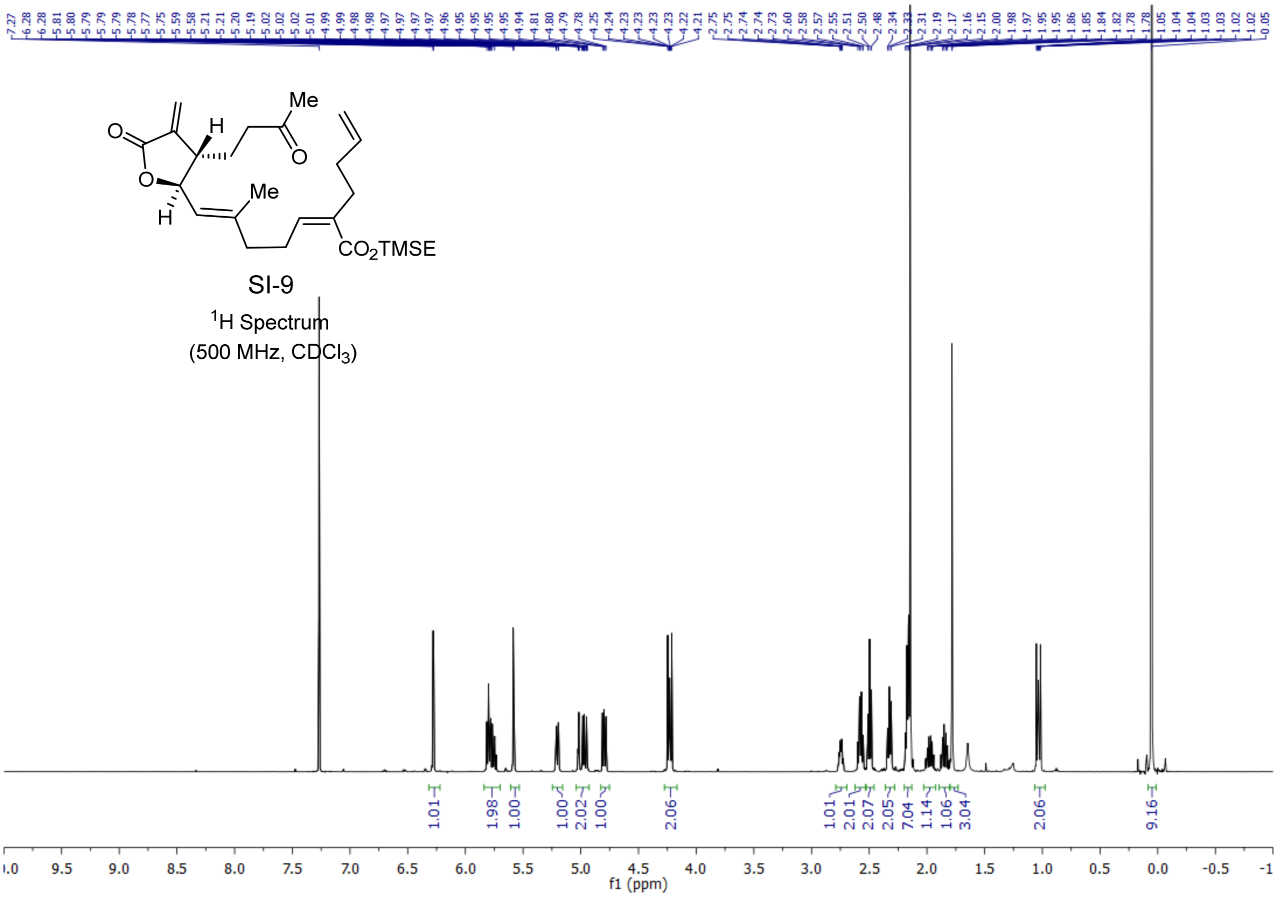

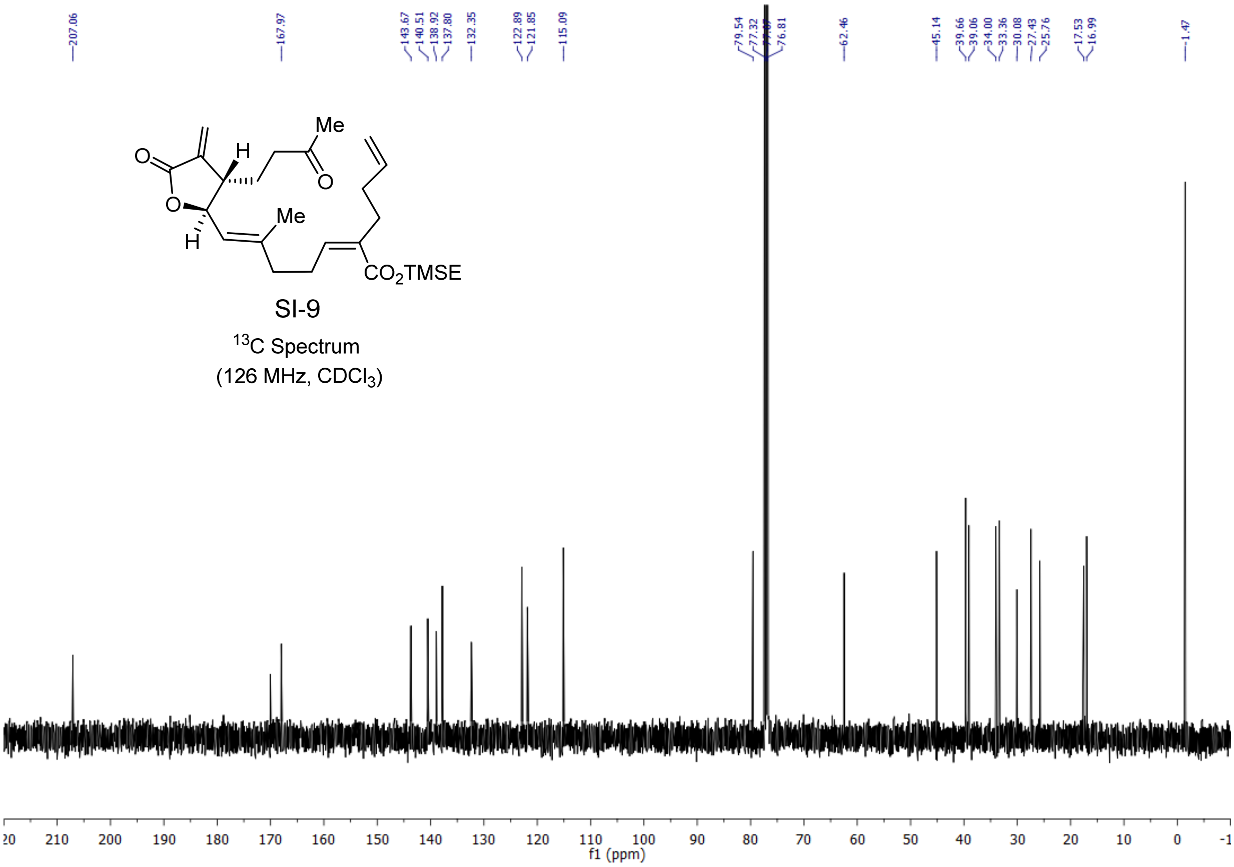

^{1}H & ^{13}C NMR Spectra for **SI-3**

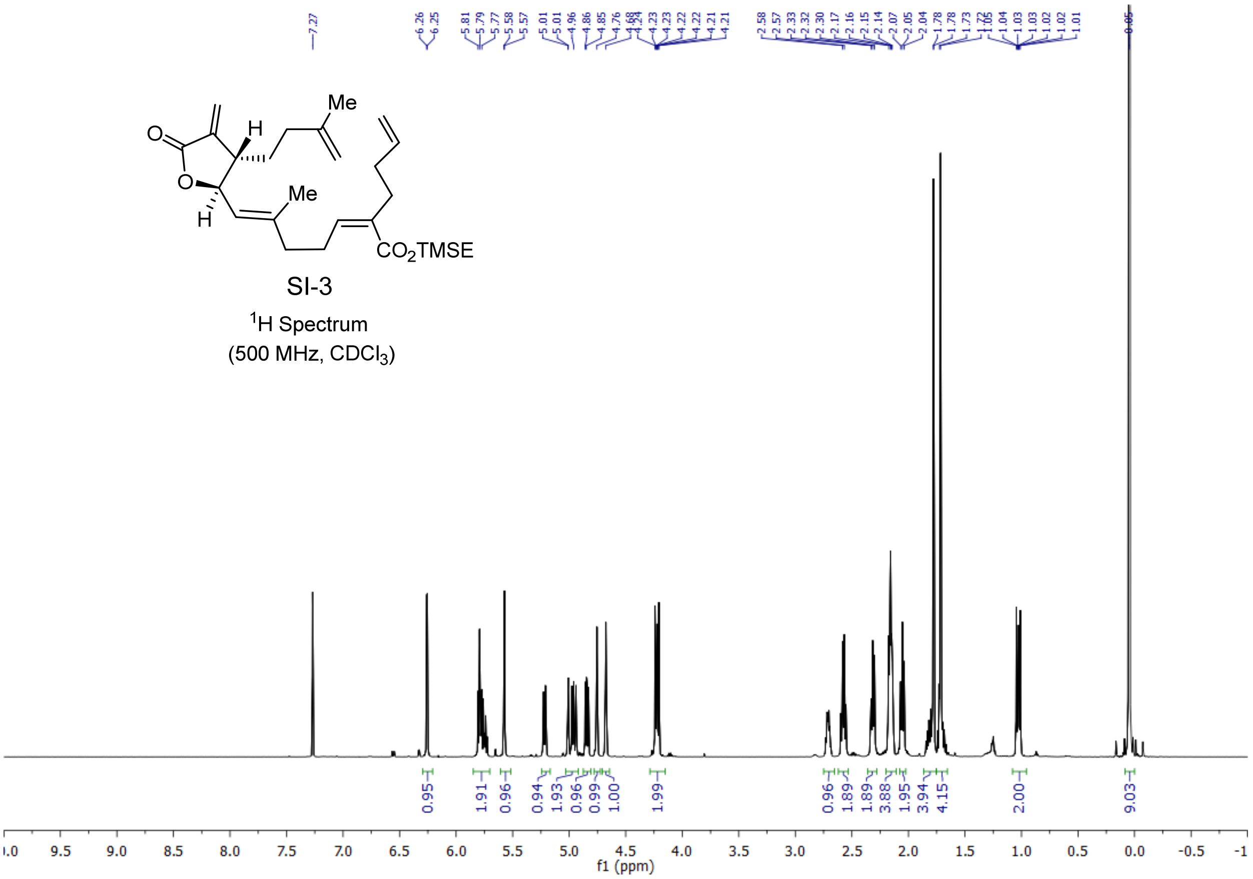

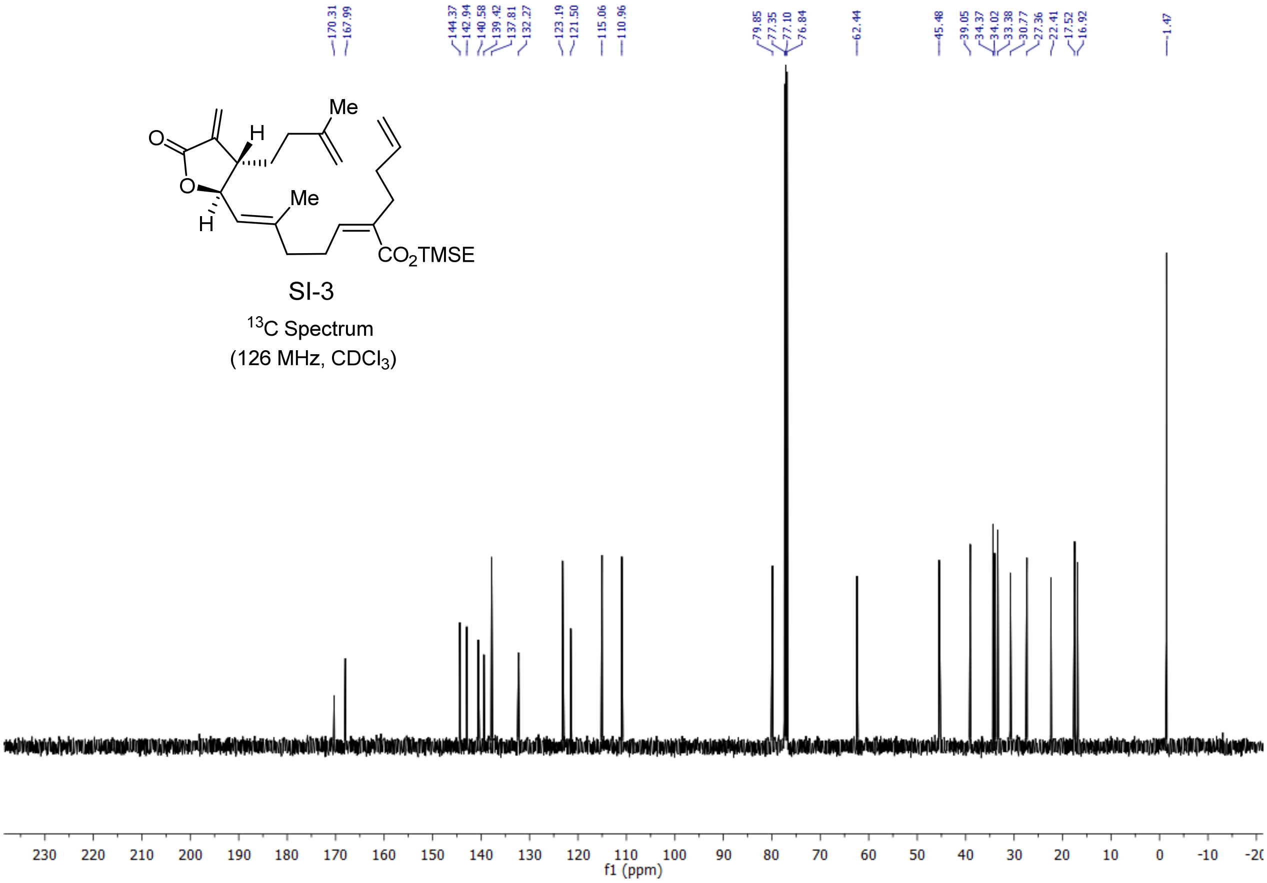

^{1}H & ^{13}C NMR Spectra for **SI-10**

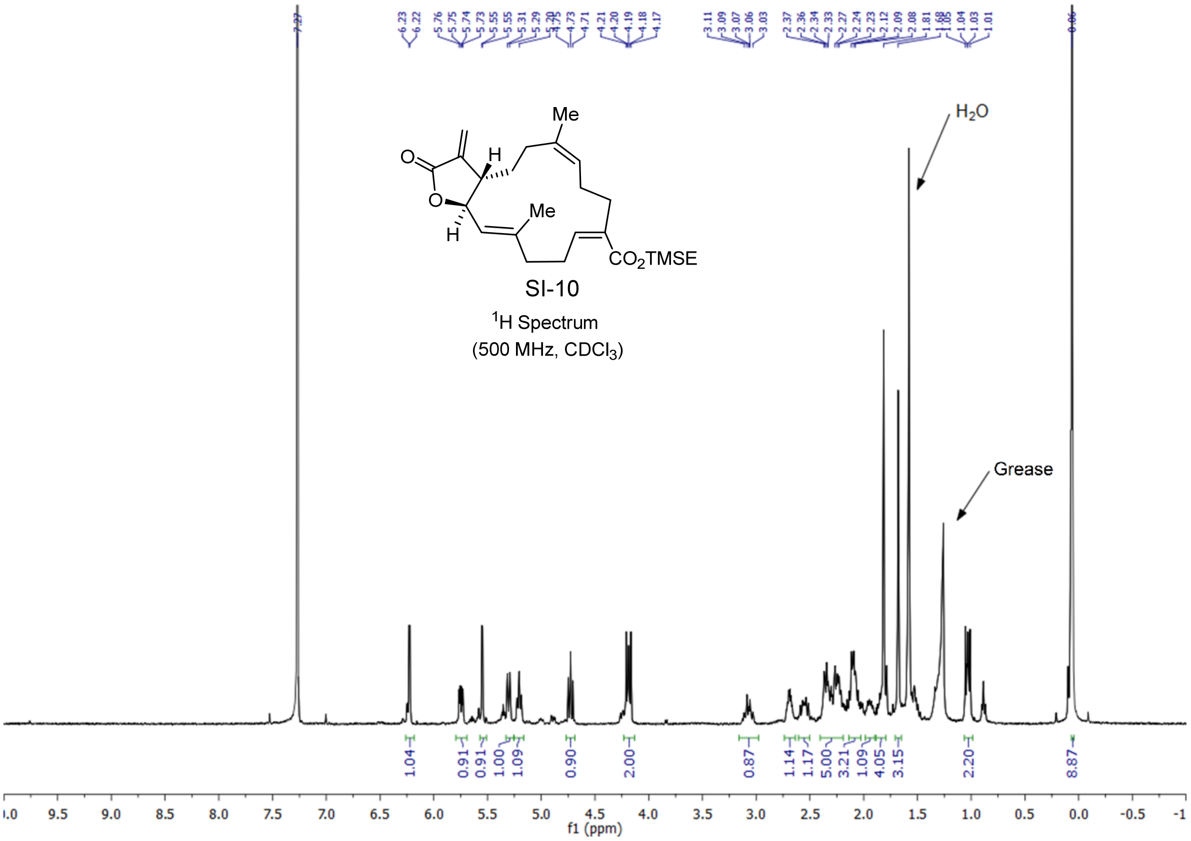

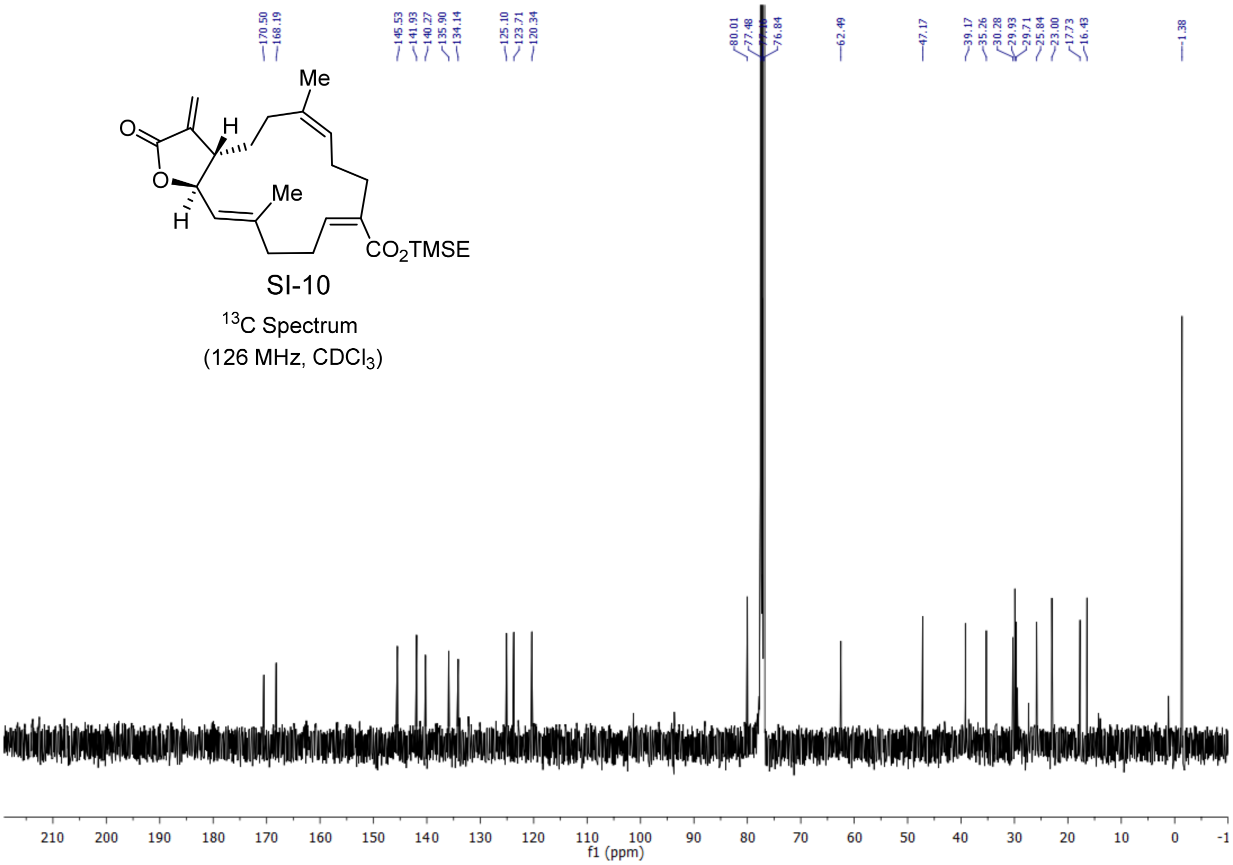

^{1}H & ^{13}C NMR Spectra for **SI-11**

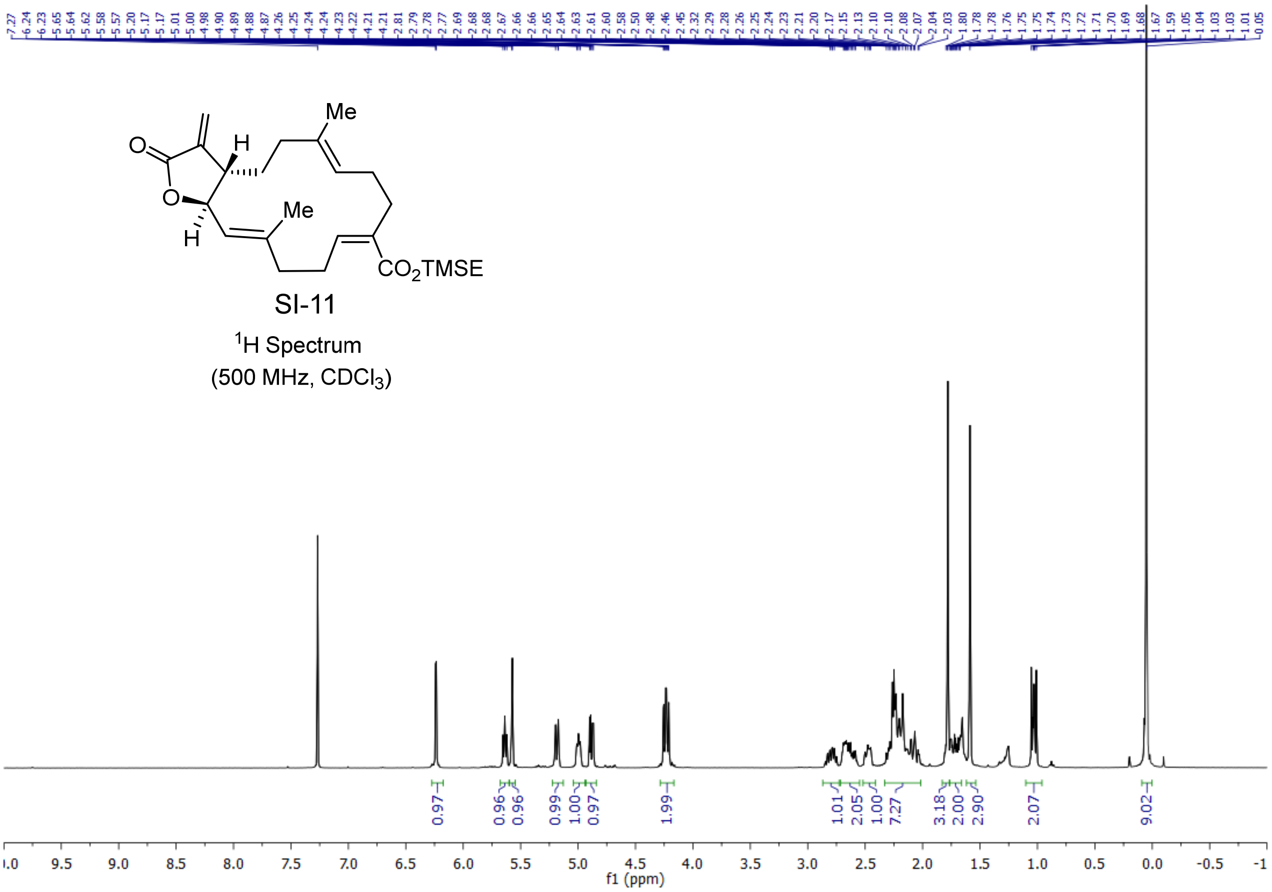

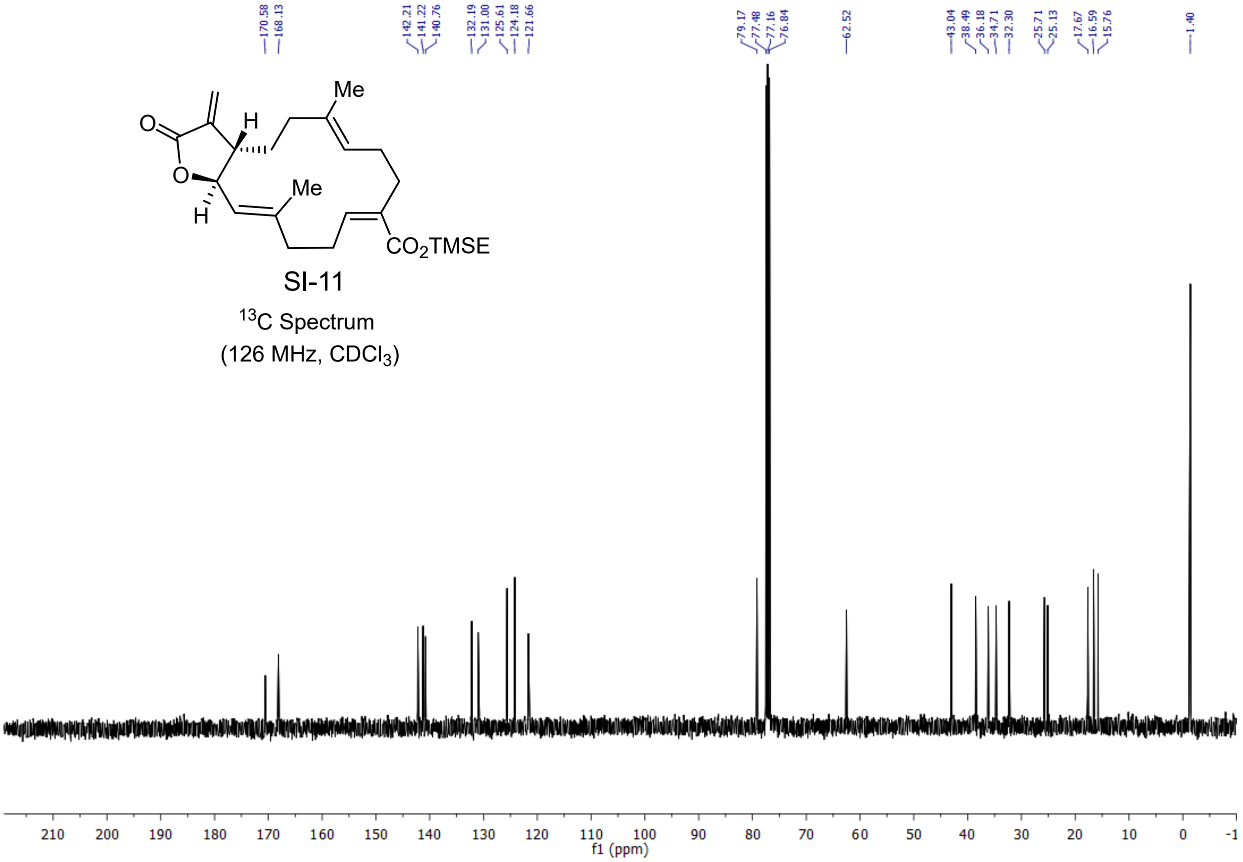

^{1}H & ^{13}C NMR Spectra for **(−)-anisomelic acid (SI-1)**

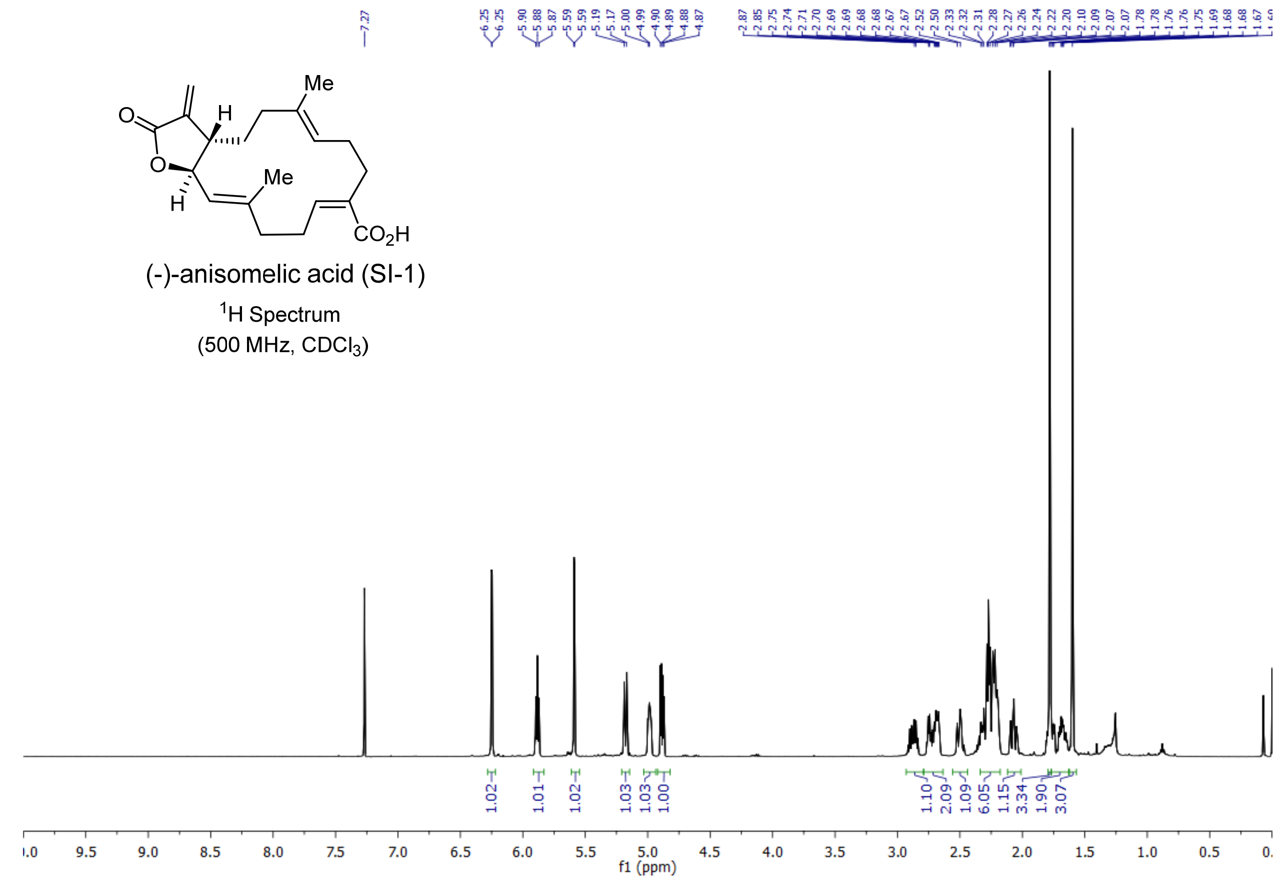

(-)-anisomelic acid (SI-1)
1H Spectrum
(500 MHz, CDCl3)
Me
Me
O
O
H
H
CO2H
7.27
6.25 6.25 5.90 5.88 5.87 5.59 5.59 5.19 5.17 5.00 4.99 4.90 4.89 4.88 4.87
2.87 2.85 2.75 2.74 2.71 2.70 2.69 2.69 2.68 2.68 2.67 2.67 2.52 2.50 2.33 2.32 2.31 2.28 2.27 2.26 2.24 2.22 2.20 2.10 2.09 2.07 2.07 1.78 1.78 1.76 1.76 1.75 1.69 1.68 1.68 1.67
1.02 1.01 1.02 1.03 1.03 1.00 1.10 2.09 1.09 6.05 1.15 3.34 1.90 3.07
f1 (ppm)

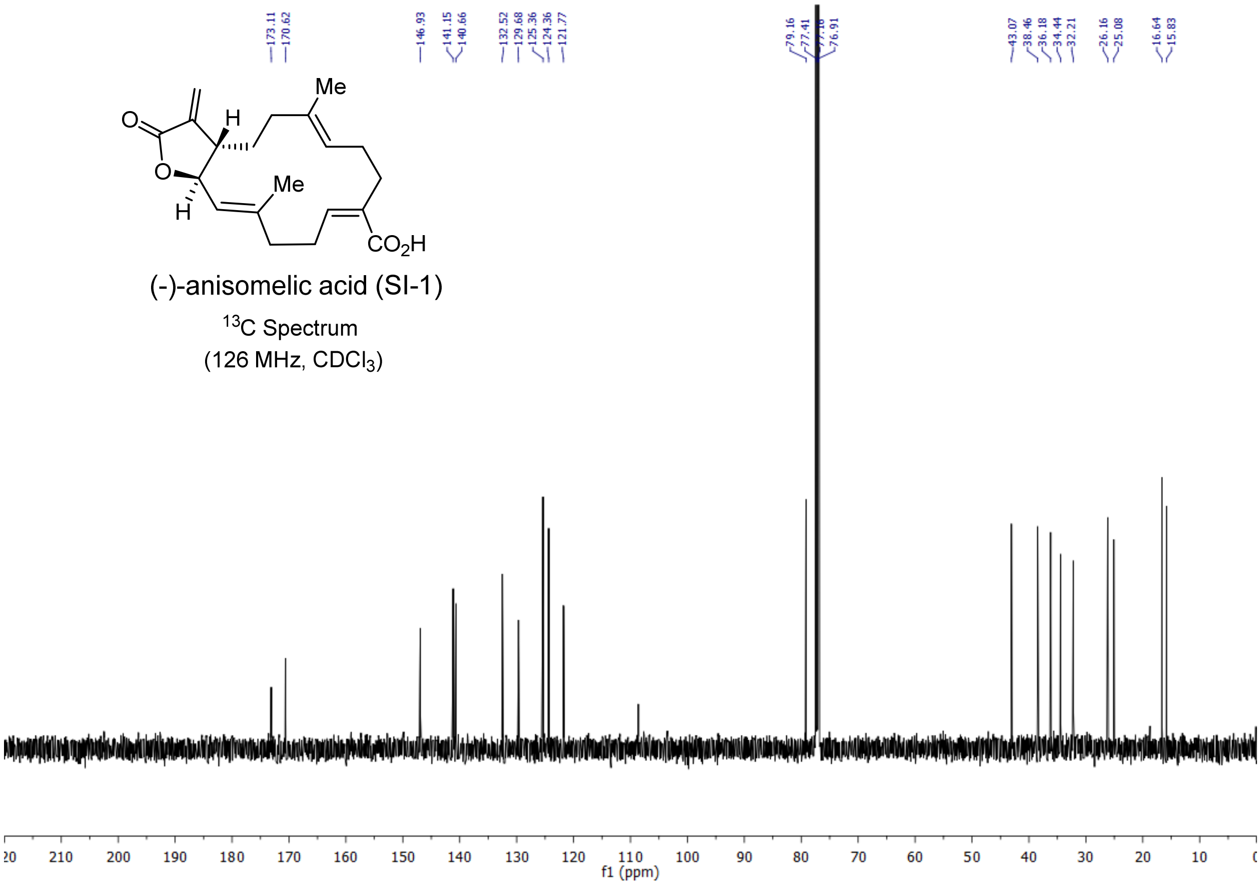

(-)-anisomelic acid (SI-1)
13C Spectrum
(126 MHz, CDCl3)
Me
Me
O
O
H
H
CO2H
173.11 170.62 146.93 141.15 140.66 132.52 129.68 125.36 124.36 121.77
79.16 77.41 76.91
43.07 38.46 36.18 34.44 32.21 26.16 25.08 16.64 15.83
f1 (ppm)

Single Crystal XRD Data

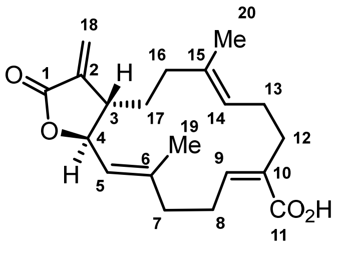

(-)-anisomelic acid (SI-1)

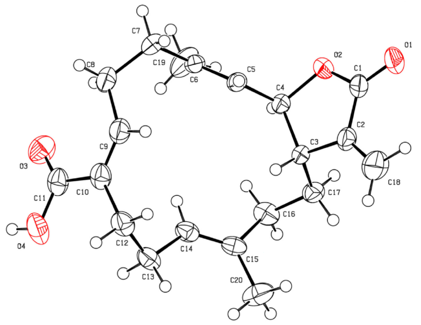

Table 1 Crystal data and structure refinement for SI-1

Identification code	exp_2868
Empirical formula	$C_{20}H_{26}O_4$
Formula weight	330.41
Temperature/K	150.00(10)
Crystal system	monoclinic
Space group	$P2_1$
a/Å	9.2505(2)
b/Å	9.14199(19)
c/Å	10.7906(2)
α/°	90
β/°	99.791(2)
γ/°	90
Volume/Å^3	899.25(3)
Z	2
ρ_{calc}g/cm^3	1.220
μ/mm^{-1}	0.674
F(000)	356.0
Crystal size/mm^3	0.13 × 0.1 × 0.08
Radiation	Cu Kα (λ = 1.54184)
2Θ range for data collection/°	8.316 to 148.164

Index ranges	$-11 \leq h \leq 11$, $-10 \leq k \leq 10$, $-12 \leq l \leq 13$
Reflections collected	4456
Independent reflections	2671 [R_{int} = 0.0197, R_{sigma} = 0.0283]
Data/restraints/parameters	2671/1/229
Goodness-of-fit on F^2	1.066
Final R indexes [I>=2σ (I)]	R_1 = 0.0370, wR_2 = 0.0937
Final R indexes [all data]	R_1 = 0.0379, wR_2 = 0.0951
Largest diff. peak/hole / e Å^{-3}	0.13/-0.19
Flack parameter	0.2(3)

Table 2 Fractional Atomic Coordinates (×10^4) and Equivalent Isotropic Displacement Parameters (Å^2×10^3) for exp_2868. U_{eq} is defined as 1/3 of the trace of the orthogonalised U_{IJ} tensor.

**Atom**	***x***	***y***	***z***	**U(eq)**
O1	11431(2)	5156(3)	6955.9(17)	70.2(7)
O2	10513.1(16)	4482(2)	5008.8(15)	48.7(5)
O3	5235(2)	5692(2)	-1332.6(16)	53.1(5)
O4	3033(2)	6119(3)	-834.7(17)	57.9(6)
C1	10375(3)	4967(3)	6150(2)	43.7(6)
C2	8806(3)	5152(3)	6217.3(19)	36.2(5)
C3	7925(2)	4646(2)	4999.1(18)	27.3(4)
C4	9098(2)	4492(2)	4143.3(18)	29.8(4)
C5	9135(2)	5711(2)	3232.3(18)	28.1(4)
C6	9189(2)	5580(2)	2012.2(18)	29.1(4)
C7	9174(2)	6914(3)	1194(2)	38.8(5)
C8	7720(3)	7067(4)	278(2)	54.4(7)
C9	6462(3)	7234(3)	979(2)	43.3(6)
C10	5064(3)	6837(3)	654(2)	39.5(5)

Atom	*x*	*y*	*z*	U(eq)
C11	4508(3)	6157(3)	-593(2)	41.5(5)
C12	4005(3)	7039(3)	1563(2)	41.4(5)
C13	3439(2)	5591(3)	2025(2)	39.6(5)
C14	4643(2)	4548(3)	2546(2)	35.3(5)
C15	4809(2)	3787(2)	3608(2)	34.0(5)
C16	6093(2)	2756(2)	3949(2)	38.6(5)
C17	7144(2)	3190(3)	5137(2)	36.1(5)
C18	8362(4)	5629(5)	7242(3)	63.3(8)
C19	9216(4)	4157(3)	1337(3)	54.9(7)
C20	3762(3)	3849(3)	4533(3)	55.3(7)

Table 3 Anisotropic Displacement Parameters (Å^2×10^3) for exp_2868. The Anisotropic displacement factor exponent takes the form: -2π^2[h^{2}a*^{2}U$_{11}$+2hka*b*U$_{12}$+…].

Atom	U_{11}	U_{22}	U_{33}	U_{23}	U_{13}	U_{12}
O1	47.2(10)	113(2)	42.7(10)	27.8(12)	-15.5(8)	-25.4(11)
O2	27.3(7)	82.0(14)	36.2(8)	13.6(9)	3.9(6)	10.0(8)
O3	64.6(11)	55.2(11)	42.2(9)	-9.2(9)	17.0(8)	-1.2(9)
O4	53.0(10)	78.0(15)	37.4(9)	-6.0(10)	-7.3(8)	23.3(10)
C1	38.9(12)	58.2(16)	31.4(11)	15.1(11)	-1.6(9)	-9.5(11)
C2	41.2(11)	42.3(13)	24.5(10)	0.5(9)	3.4(9)	-6.5(9)
C3	27.3(9)	32.1(11)	22.9(9)	2.0(8)	5.5(7)	0.2(8)
C4	28.6(9)	36.1(11)	25.2(9)	2.3(9)	5.7(8)	2.8(8)
C5	29.0(9)	29.2(10)	26.9(9)	-1.1(8)	6.7(7)	-4.1(8)
C6	26.7(9)	34.0(10)	27.7(10)	-1.9(9)	7.2(7)	-4.5(8)
C7	40.5(11)	45.0(14)	33.8(12)	6.8(10)	14.7(9)	-3.8(10)
C8	47.4(13)	85(2)	32.2(12)	17.4(14)	10.8(10)	11.1(14)
C9	45.2(13)	52.9(15)	32.0(11)	3.9(11)	7.5(10)	10.3(11)

Atom	U_{11}	U_{22}	U_{33}	U_{23}	U_{13}	U_{12}
C10	45.7(13)	37.4(13)	35.0(11)	2.3(10)	5.2(9)	13.8(10)
C11	52.4(13)	34.4(12)	36.7(12)	6.0(10)	4.6(10)	12.5(11)
C12	38.2(11)	44.6(13)	40.7(12)	-5.2(11)	4.7(9)	12.7(10)
C13	28.7(10)	47.9(13)	40.2(12)	-7.4(11)	0.4(9)	5.4(10)
C14	27.8(9)	39.0(12)	39.2(12)	-7.8(10)	6.2(8)	2.9(9)
C15	26.1(10)	29.7(11)	46.1(13)	-6.4(10)	5.9(9)	-5.6(8)
C16	37.0(11)	25.5(11)	52.0(14)	-2.3(10)	4.1(10)	-2.5(9)
C17	34.1(11)	35.2(12)	39.8(12)	8.2(10)	9.1(9)	-1.6(9)
C18	66.0(19)	90(2)	34.8(13)	-18.0(15)	11.6(12)	-8.1(17)
C19	86(2)	45.3(16)	40.2(14)	-13.7(12)	29.2(13)	-11.2(13)
C20	52.9(16)	47.5(15)	73.2(19)	10.9(14)	32.4(14)	0.5(12)

Table 4 Hydrogen Atom Coordinates (Å×10^4) and Isotropic Displacement Parameters (Å^2×10^3) for exp_2868.

Atom	*x*	*y*	*z*	U(eq)
H4	2757.86	5847.35	-1560.77	87
H3	7207.57	5395.41	4662.62	33
H4A	8963.57	3560.68	3689.1	36
H5	9119.95	6656.54	3547.35	34
H7A	9973.81	6850.44	719.93	47
H7B	9329.31	7777.67	1721.47	47
H8A	7765.3	7914.05	-255.21	65
H8B	7565.3	6208.53	-256.23	65
H9	6693.87	7683.84	1760.62	52
H12A	3174.21	7610.46	1156.14	50
H12B	4487.61	7591	2283.75	50

Table 4 Hydrogen Atom Coordinates (Å×10^4) and Isotropic Displacement Parameters (Å^2×10^3) for exp_2868.

**Atom**	***x***	***y***	***z***	**U(eq)**
H13A	2857.03	5800.87	2671.89	47
H13B	2802.04	5123.48	1330.89	47
H14	5370.89	4417.28	2058.35	42
H16A	6632.92	2715.64	3253.8	46
H16B	5720.46	1781.91	4062.35	46
H17A	7872.81	2426.06	5344.88	43
H17B	6600.32	3266.61	5828.34	43
H18A	7330(40)	5700(40)	7290(30)	60(9)
H18B	9130(40)	5900(40)	7970(30)	66(9)
H19A	9450.19	3382.06	1938.04	82
H19B	9943.41	4195.31	800.68	82
H19C	8270.24	3977.45	837.26	82
H20A	2929.56	4438.39	4194.53	83
H20B	4248.82	4270.25	5307.87	83
H20C	3441.01	2877.46	4687.54	83

References

[1] Li Y, Cao L and Li G *et al.* Remdesivir Metabolite GS-441524 Effectively Inhibits SARS-CoV-2 Infection in Mouse Models. *J Med Chem* 2022; **65**: 2785-2793.

[2] Trott, O and Olson AJ. AutoDock Vina: Improving the Speed and Accuracy of Docking with a New Scoring Function, Efficient Optimization and Multithreading. *J Comp Chem* 2010; **31**: 455-461.

[3] DeLano WL. The PyMOL Molecular Graphics System. Palo Alto, CA: DeLano Scientific; 2002.

[4] *Summary on Compassionate Use: Remdesivir Gilead*; European Medicines Agency, European Union. 2020.

[5] (a) Arisawa M, Nimura A and Ikeda T. *et al*. Biologically Active Macrocyclic Diterpenoids from Chinese Drug "Fáng Féng Cáo" I. Isolation and Structure1. *Planta Medica* 1986; **52**: 38-41. (b) Toubiana R, Toubiana, MJ and McPhail AT. Structures and conformations of the fourteen-membered ring diterpene ovatodiolide and its acid cyclization product: nuclear Overhauser effect studies in solution and X-ray crystal structure analyses of ovatodiolide and ovatodiolic acid. *J Chem Soc Perkin Transactions* 1976; **2**: 1881-1889.

[6] Chausset-Boissarie, Àrvai LR and Cumming, GR *et al*. Asymmetric synthesis of (+)-vertine and (+)-lythrine. *Org Biomol Chem* 2012; **10**: 6473-6479.

[7] Arisawa M, Nimura M and Ikeda, A *et al*. Biologically Active Macrocyclic Diterpenoids from Chinese Drug "Fáng Féng Cáo" I. Isolation and Structure. *Planta Med* 1986; **52**: 38-41.

[8] Xiang J, Ding Y and Li J *et al*. Ovatodiolides: Scalable Protection-Free Syntheses, Configuration Determination, and Biological Evaluation against Hepatic Cancer Stem Cells. *Angew Chem Int Ed* 2019; **131**: 10697-10700.
